# ACH2.0/E, the Consolidated Theory of Conventional and Unconventional Alzheimer’s Disease: Origins, Progression, and Therapeutic Strategies

**DOI:** 10.3390/ijms25116036

**Published:** 2024-05-30

**Authors:** Vladimir Volloch, Sophia Rits-Volloch

**Affiliations:** 1 Department of Developmental Biology, Harvard School of Dental Medicine, Boston, MA 02115, USA; 2 Division of Molecular Medicine, Children’s Hospital, Boston, MA 02115, USA; 3 Department of Biological Chemistry and Molecular Pharmacology, Harvard Medical School, Boston, MA 02115, USA

**Keywords:** Conventional and unconventional Alzheimer’s disease, Alzheimer’s disease-like dementia (ADLD) and Alzheimer’s disease-related dementia (ADRD), Neuronal integrated stress response (ISR), Intraneuronal Aβ (*i*Aβ) and AβPP-independent generation of *i*Aβ, Amyloid cascade hypothesis 2.0 (ACH2.0) and expanded amyloid cascade hypothesis 2.0 (ACH2.0/E), Therapeutic strategies for conventional and unconventional Alzheimer’s disease and for aging-associated cognitive decline, Traumatic brain injury (TBI) and Alzheimer’s disease, Chronic traumatic encephalopathy (CTE) and Alzheimer’s disease, Viral and bacterial infection and Alzheimer’s disease, Neuroinflammation and systemic inflammation and Alzheimer’s disease

## Abstract

The centrality of amyloid-beta (Aβ) is an indisputable tenet of Alzheimer’s disease (AD). It was initially indicated by the detection (1991) of a mutation within Aβ protein precursor (AβPP) segregating with the disease, which served as a basis for the long-standing Amyloid Cascade Hypothesis (ACH) theory of AD. In the intervening three decades, this notion was affirmed and substantiated by the discovery of numerous AD-causing and AD-protective mutations with all, without an exception, affecting the structure, production, and intraneuronal degradation of Aβ. The ACH postulated that the disease is caused and driven by extracellular Aβ. When it became clear that this is not the case, and the ACH was largely discredited, a new theory of AD, dubbed ACH2.0 to re-emphasize the centrality of Aβ, was formulated. In the ACH2.0, AD is caused by physiologically accumulated intraneuronal Aβ (*i*Aβ) derived from AβPP. Upon reaching the critical threshold, it triggers activation of the autonomous AβPP-independent *i*Aβ generation pathway; its output is retained intraneuronally and drives the AD pathology. The bridge between *i*Aβ derived from AβPP and that generated independently of AβPP is the neuronal integrated stress response (ISR) elicited by the former. The ISR severely suppresses cellular protein synthesis; concurrently, it activates the production of a small subset of proteins, which apparently includes components necessary for operation of the AβPP-independent *i*Aβ generation pathway that are absent under regular circumstances. The above sequence of events defines “conventional” AD, which is both caused and driven by differentially derived *i*Aβ. Since the ISR can be elicited by a multitude of stressors, the logic of the ACH2.0 mandates that another class of AD, referred to as “unconventional”, has to occur. Unconventional AD is defined as a disease where a stressor distinct from AβPP-derived *i*Aβ elicits the neuronal ISR. Thus, the essence of both, conventional and unconventional, forms of AD is one and the same, namely autonomous, self-sustainable, AβPP-independent production of *i*Aβ. What distinguishes them is the manner of activation of this pathway, i.e., the mode of causation of the disease. In unconventional AD, processes occurring at locations as distant from and seemingly as unrelated to the brain as, say, the knee can potentially trigger the disease. The present study asserts that these processes include traumatic brain injury (TBI), chronic traumatic encephalopathy, viral and bacterial infections, and a wide array of inflammatory conditions. It considers the pathways which are common to all these occurrences and culminate in the elicitation of the neuronal ISR, analyzes the dynamics of conventional versus unconventional AD, shows how the former can morph into the latter, explains how a single TBI can hasten the occurrence of AD and why it takes multiple TBIs to trigger the disease, and proposes the appropriate therapeutic strategies. It posits that yet another class of unconventional AD may occur where the autonomous AβPP-independent *i*Aβ production pathway is initiated by an ISR-unrelated activator, and consolidates the above notions in a theory of AD, designated ACH2.0/E (for expanded ACH2.0), which incorporates the ACH2.0 as its special case and retains the centrality of *i*Aβ produced independently of AβPP as the driving agent of the disease.

In lieu of introduction and to better orient the reader, the following is a rationalization of some of the key terms utilized in the present study. The major attribute of the ACH is the centrality of Aβ in Alzheimer’s disease. When predictions made by the ACH could not be verified, and the theory could not be validated, it was replaced by another theory of AD that retained the centrality of Aβ as its major attribute and a common denominator. It seemed to us reasonable, therefore, to designate this new theory as ACH2.0. Since its introduction, the ACH2.0 has been analyzed and characterized in numerous publications [1,2,3,4,5,6,7] and by now this term should be familiar. The present study introduces a conceptual development of the ACH2.0. The new generalized theory incorporates the ACH2.0 as the integral part and takes its central tenets one or two steps further; it also preserves the centrality of Aβ as its defining feature. It constitutes the expansion of the ACH2.0 and it is rational therefore to designate it ACH2.0/E (for expanded ACH2.0). These designations are suitable for also another reason: both ACH2.0 and ACH2.0/E are firmly grounded in the empirical data but are no less hypothetical than the initial ACH; after all, “ACH” stands for Amyloid Cascade Hypothesis. Yet both, ACH2.0 and ACH2.0/E, make well-defined and verifiable predictions, and the present study outlines a clear path to their validation.

The available literature describes numerous conditions that closely resemble Alzheimer’s disease. These conditions are referred to as “AD-like dementia” (ADLD) or “AD-related dementia” (ADRD). The present study asserts that many, if not the majority of the ADLD and ADRD cases constitute a discrete bona fide class of AD triggered by distinct signaling pathways. As described in the present study, this class of Alzheimer’s disease differs from the ACH2.0-defined category of AD not only in its origins, i.e., the manner of its causation, but, equally if not more importantly, also in the applicable therapeutic strategies. These two classes of AD should be, therefore, distinguishable in their designations. In the ACH2.0, as in the ACH, the disease is caused by AβPP-derived Aβ. In view of the conventionality of its Aβ-driven causation, we designated this class of Alzheimer’s disease as “conventional” AD. Accordingly, since cases that comprise the second class of Alzheimer’s disease are other than “conventional” AD, it seems reasonable to designate them “unconventional” AD. These designations are used consistently throughout the present article.

## 1. The ACH: A Proposition in Distress

### 1.1. The Definition

In 1992, a theory of AD, designated the “Amyloid Cascade Hypothesis” (ACH), was proposed [8]. Its authors defined it as follows: “Our hypothesis is that deposition of amyloid β protein, the main component of the plaques, is the causative agent of Alzheimer’s pathology and that the neurofibrillary tangles, cell loss, vascular damage, and dementia follow as the direct result of this deposition” [8]. The occurrence of the Aβ-containing plaques has been established long before the inauguration of the ACH. What gave this theory credence was the discovery, in the preceding year, of an AβPP mutation affecting the production of Aβ and segregating with, and apparently causing, the early onset of the disease (familial AD, FAD) [9]. At the time of its inauguration, the ACH theory of AD appeared to be consistent with the accumulated data, and was widely accepted. Consequently, it became a foundation for experimental research in the field for many years to come; it guided the design of the candidate AD drugs and informed construction of transgenic animal models of the disease [10].

### 1.2. The Inconsistencies

The inconsistencies within the ACH manifested when the initial transgenic models of AD were developed and constructed. In these models, human Aβ is produced from multiple, several dozens, of transgenes. It is acutely overexpressed and over-secreted, resulting in extracellular depositions equal to or exceeding those occurring in AD patients. Yet, only limited symptoms consistent with AD were observed, and the principal hallmark of the disease, neurofibrillary tangles (NFT), was not detected in these models. To address this issue, multiple FAD mutations were introduced into transgenic animal models. Those included not only mutations of Aβ and AβPP, but also FAD mutations of presenilins (PSEN). These modifications, however, affected mainly the kinetics of symptoms’ manifestation, i.e., changes were quantitative rather than qualitative; no NFT formation occurred. In hindsight, from our current perspective (discussed in Section 4 below), it appears that cognitive impairments observed in these models and attributed to AD, were, in fact, caused by the integrated stress response in neuronal cells, shown to occur in these models, and that their attribution to AD was not justified.

Multiple candidates for AD drugs, designed with the ACH guidance and referred to henceforth as the ACH-based drugs, were highly effective, and indeed performed rather spectacularly, in transgenic animal models of AD. They not only substantially reduced levels of extracellular Aβ, but also stopped the progression and in some cases even reversed the symptoms of the disease [11,12,13]. Such an outcome, however, was not the case in AD patients. In multiple human clinical trials, the ACH-based AD drugs failed no less spectacularly than they succeeded in animal models; no efficacy whatsoever was observed [14,15] (the apparent exceptions, the marginal beneficial effects of ACH-based drugs lecanemab and donanemab, are consistent with the preceding statement and are interpreted in detail in Section 12 below).

### 1.3. Extracellular Aβ Is Apparently Not the Causative Agent of Alzheimer’s Disease

Importantly, the reason that the ACH-based AD drugs failed in human clinical trials was not because they were inefficient mechanistically. On the contrary, they performed their mechanistic mission no less efficiently than they did in transgenic animal models of AD. These drugs were designed to reduce the levels of extracellular Aβ, and this is precisely what they did, with great efficiency, in AD patients, causing, in a dose-dependent manner, the up to 80% reduction in the level of Aβ in CSF [14,15]. The outcomes of human clinical trials of the ACH-based drugs strongly indicated that extracellular Aβ is not the causative agent of Alzheimer’s disease. This conclusion was corroborated by the observations of a rather poor correlation between the levels of extracellular Aβ depositions and the occurrence of AD. Indeed, in a substantial fraction (over one third) of the aged general human population, the levels of extracellular Aβ depositions are either equal to or exceed those observed in typical AD patients, yet no cognitive impairments manifest, nor do neurodegenerative problems occur [16,17,18,19,20,21,22]. The converse is also true: in some AD patients, the disease develops despite the lack of the excessive extracellular Aβ depositions [23]. Cumulatively, the above arguments strongly suggest that extracellular Aβ is not the cause of AD.

## 2. The ACH2.0 Theory of Conventional AD

### 2.1. The Centrality of Aβ in AD: Intraneuronal Aβ (iAβ)

As mentioned above, the discovery of an AβPP mutation segregating with and apparently causing AD was the immediate cause for formulation of the ACH. In the more than three decades that followed, many more mutations that cause AD [24], and one that protects from it [25,26], were discovered. These mutations occur within Aβ, within AβPP region adjacent to the gamma-cleavage sites, or within the precinilins. They all, invariably, affect the structure, production, and intraneuronal degradation of Aβ, and with one exception, they all cause the early onset of the disease. The exception in question is the Icelandic mutation [25,26]. It changes a single nucleotide and replaces a single amino acid residue within Aβ, and this substitution is sufficient to confer to the carriers of this mutation protection from both AD and Aging-Associated Cognitive Decline (AACD) [25]. The observed uniformity and invariability of the effect of all known AD-causing and AD-protective mutations on production, structure, and intraneuronal degradation of Aβ constitutes a convincing argument for the notion of the centrality and causative role of Aβ in Alzheimer’s disease. Since extracellular Aβ can be excluded as the cause of AD, the role of the causative agent of the disease falls to another category of Aβ: physiologically occurring intraneuronal Aβ (*i*Aβ). This conclusion is strongly supported by multiple investigations, which indicate that *i*Aβ correlates with AD much better than its extracellular counterpart and appears to be the major constituent of the disease [27,28,29,30,31,32,33,34,35,36,37,38,39].

### 2.2. Physiological Origins of AβPP-Derived iAβ

Physiologically, Aβ is generated as a segment embedded within its precursor, AβPP. It is released by two proteolytic cleavages. The first cleavage is enacted by beta-secretase (BACE: Beta-site AβPP Cleaving Enzyme). This cleavage takes place between residues 671 and 672 of AβPP. One of the two resulting products is the C-terminal fragment (CTF) of AβPP. It contains Aβ as its N-terminal segment and consists of 99 amino acid residues, and hence is dubbed C99. The C99 fragment is further cleaved by gamma-secretase, thus releasing Aβ. The gamma-cleavage occurs at variable positions within a short segment of C99 and results in Aβ with distinct C-termini and of variable length, usually 40 or 42 amino acid residues. Both beta and gamma cleavages take place on cellular membranes. The gamma cleavage typically occurs on the plasma membrane, and the newly generated Aβ is secreted. A small fraction of gamma cleavages occur not on the plasma membrane, but rather on the intracellular membranes within various cellular organelles [40,41,42,43,44,45,46,47,48]. Crucially, Aβ produced on intracellular membranes is retained intraneuronally. The retention of Aβ generated on intracellular membranes constitutes one origin source of AβPP-derived *i*Aβ. The second origin source is the cellular uptake of secreted Aβ [49,50,51,52,53,54]. This, apparently, requires the oligomerization of Aβ [53,54] and occurs via an array of cellular receptors [55,56,57,58,59,60,61,62,63]. Importantly, both processes contributing to the pool of AβPP-derived *i*Aβ, namely its intraneuronal retention and its importation from the extracellular space, occur physiologically.

### 2.3. The Principal Tenets of the ACH2.0

Clinical trials of the candidate AD drugs constituted the pivotal point in the development of the Alzheimer’s field, and their results provided the rationale for formulation of the ACH2.0. They also provided a basis for defining the principal attributes of this theory of AD. In these trials, a demonstrated substantial reduction in the levels of extracellular Aβ in symptomatic AD had no beneficial effect whatever [14,15]. This suggests that extracellular Aβ neither causes nor drives the disease. Since another independent set of data, discussed above, strongly indicates that Aβ both initiates and powers the disease [24,25,26], and since another intraneuronal pool of *i*Aβ does occur [27,28,29,30,31,32,33,34,35,36,37,38,39], it has to be the latter, which serves as the disease-causative and -driving agent. On the other hand, since the substantial inhibition (by BACE1 inhibitors) of the generation of Aβ by the proteolysis of AβPP also had no efficacy whatsoever in symptomatic AD [14,15], it can be concluded that in the disease, Aβ is generated independently of AβPP and is retained within the neurons as *i*Aβ. These two attributes, i.e., the causative role of intraneuronal Aβ in the disease and its generation independently of AβPP followed by its subsequent intraneuronal retention, constitute the principal tenets of the ACH2.0 [1,2,3,4,5,6,7]. As further discussed below, conventionally, the disease is caused by AβPP-derived *i*Aβ accumulated over the critical threshold, thus triggering the activation of the AβPP-independent pathway of the generation of *i*Aβ, which drives the AD pathology. Thus, in the ACH2.0 paradigm, the disease is caused and driven by *i*Aβ derived differentially in two distinct pathways: AβPP-derived *i*Aβ cannot reach the disease-driving levels, but triggers AD, whereas *i*Aβ generated independently of AβPP propels the AD pathology and, as discussed below, perpetuates its own production [1,2,3,4,5,6,7]. 

## 3. Mechanistic Aspects of AD in the ACH2.0 Perspective

### 3.1. At Sufficient Levels, AβPP-Derived iAβ Mediates Activation of the eIF2α Kinases PKR and HRI; Phosphorylation of eIF2α Elicits the Neuronal Integrated Stress Response

Provided AβPP-derived *i*Aβ reaches a certain level, referred to above as “the critical threshold”, it triggers activation of PKR and HRI, both kinases of eukaryotic translation initiation factor 2 alpha, eIF2α [64,65,66,67,68,69,70]. Multiple studies demonstrated the connection between Aβ and the activity of the PKR kinase. The concurrent presence of both PKR and phosphorylated eIF2α was shown in cells and animal model systems overexpressing Aβ [64,65,66]. Crucially, the activated PKR was demonstrated in neurons of AD patients [67,68]. Apparently, there are two *i*Aβ-mediated pathways to activate the PKR kinase. One pathway involves TNFα [69], whereas another proceeds via PKR activator (designated PACT) [70]. The utilization of the latter in Alzheimer’s disease is indicated by the colocalization of both PACT and activated PKR in the affected neurons of Alzheimer’s patients [70].

On the other hand, the *i*Aβ-triggered activation of the HRI kinase in the AD-affected neurons is a corollary of the mitochondrial dysfunction. Mitochondrial distress in AD-affected neurons is one of the earliest manifestations of the disease, and its link to *i*Aβ was extensively studied and is well established [71,72,73,74,75,76,77,78,79,80,81,82,83,84,85,86,87,88]. Mitochondrial dysfunction has multiple significant implications for cellular physiology. Among them is its capacity to activate the HRI kinase. Since HRI is located in the cytosol, the process of its activation by mitochondrial dysfunction requires transfer of a signal across the mitochondrial membrane. This is effected, apparently, by two mitochondrial proteins [89,90]. One is OMA1, a mitochondrial protease activated by mitochondrial dysfunction. Upon its activation, OMA1 cleaves the second mitochondrial protein involved in signal transduction, DELE1. Following the cleavage, one of the DELE1 fragments is transported across the mitochondrial membrane into the cytosol where it binds to and activates the HRI kinase [89,90]. The levels of *i*Aβ, the “critical threshold”, required to trigger the activation of PKR could be and probably are different from those needed to initiate the activation of HRI. This, however, is inconsequential because either kinase would, upon activation, phosphorylate eIF2α and thus elicit the integrated stress response, ISR.

### 3.2. The ISR: Transcriptional and Translational Cellular Reprogramming

The integrated stress response is a signaling pathway, which is conserved evolutionary and is elicited by, and activated in response to, various pathological and environmental stimuli [91,92,93,94,95,96,97,98,99,100]. The latter include a great variety of cellular stresses, inflammation, viral and bacterial infections, protein aggregation and improper folding, defects in protein homeostasis, and nutrient deprivation. The ISR is “integrated” because of the uniformity of its reaction to a variety of stimuli. The mechanistic underpinning of this uniformity is the convergence of all inducements on the single event, namely the site-specific phosphorylation of eIF2α at the serine residue 51. This phosphorylation event is effected by one of the four members of the family of eIF2α kinases: PERK (PKR-like ER kinase), PKR (protein kinase double-stranded RNA-dependent), GCN2 (general control non-derepressible-2), and HRI (heme-regulated inhibitor). When any one of these kinases is activated, eIF2α is phosphorylated, and the ISR ensues. The consequences of the ISR for the cellular physiology are rather dramatic. The general landscapes of both cellular transcription and translation are radically transformed and reprogrammed [91,92,93,94,95,96,97,98,99,100]. On the transcription side, a number of new transcription factors are activated. On the cellular translation side, the total protein synthesis is severely suppressed. Concurrently, this suppression is accompanied by the activation of translation, mostly in a cap-independent manner, of a small subset of cellular mRNA species.

### 3.3. The ISR Provides “Missing” Essential Components and Enables Operation of the AβPP-Independent Pathway of iAβ Generation

Thus, in Alzheimer’s disease, *i*Aβ-triggered activation of the PKR and/or HRI kinases results in phosphorylation of eIF2α and elicitation of the neuronal integrated stress response. The latter is the pivotal event. This is because the small subset of cellular proteins, newly translated under the ISR conditions, presumably includes one or more essential components that are required for operation of the AβPP-independent *i*Aβ generation pathway, but which are “missing” under the regular cellular circumstances. With the “missing” components available, the AβPP-independent *i*Aβ production pathway is activated and AD commences [1,2,3,4,5,6,7]. Importantly, the progression, in fact even the occurrence, of the disease depends upon the continuous operation of the AβPP-independent pathway of *i*Aβ generation. Consequently, it depends upon the continuous availability of the required components of the pathway, which, in turn, depends upon the sustained maintenance of the ISR conditions in neuronal cells [1,2,3,4,5,6,7].

## 4. The AβPP-Independent Pathway of *i*Aβ Generation

### 4.1. AβPP-Independent iAβ Production Pathway Is Essential in AD, the Disease Commences Only If and When the Pathway Is Sustainably Activated

In the ACH2.0 paradigm, sustainable operation of the AβPP-independent pathway is essential for the occurrence and progression of AD. This pathway is, in fact, the active and crucial core of the disease [1,2,3,4,5,6,7]. This is because production and accumulation of *i*Aβ in the AβPP proteolytic pathway is apparently inadequate in reaching the AD pathology-driving levels required for progression of the disease [1,2,3,4,5,6,7]. As discussed below and elsewhere [4,7], the efficiency of the AβPP-independent *i*Aβ generation pathway is substantially, possibly orders of magnitude, greater than that of the AβPP proteolytic pathway and thus is sufficient (and necessary) to support the progression of AD. With the former inoperative, AD simply cannot occur. 

This point, the indispensability of the operational AβPP-independent *i*Aβ production pathway in AD can be vividly illustrated by the inadequacy of the current transgenic animal models of the disease [7]. In these models, Aβ is produced in the AβPP proteolytic pathway from dozens of human transgenes, and its extracellular deposition significantly exceeds that seen in AD. Transgenic animals exhibit AD-related symptoms, yet the full spectrum of the AD pathology cannot be attained; no formation of neurofibrillary tangles was observed in these models. In these model systems, AβPP-derived *i*Aβ accumulates by the two mechanisms described above, intraneuronal retention and importation of secreted Aβ. As discussed above, it was shown to trigger the activation of PKR and the phosphorylation of eIF2α. This elicits the neuronal ISR and triggers cognitive impairments such as deficits in neuronal plasticity, long-term memory formation, and learning, all processes requiring new protein synthesis, which is suppressed by the ISR [101,102,103,104,105,106,107,108,109,110,111,112,113,114,115,116]. When the ISR is prevented in these models by genetic manipulations or pharmacological treatment, so are the cognitive impairments; when the ISR is suppressed by a small-molecule ISR inhibitor ISRIB, the cognitive impairments are abrogated [117,118,119,120,121,122,123,124,125]. Thus, the cognitive impairments observed in these models can be attributed to the effect of the neuronal ISR rather then to AD; in fact, no AD occurs in these models. This is because in these model systems, for the reasons discussed in detail in [7], the AβPP-independent *i*Aβ generation pathway is inoperative. 

### 4.2. Molecular Mechanisms Potentially Enabling Operation of the AβPP-Independent iAβ Production Pathway

#### 4.2.1. The Pivotal Role of the AUG Codon for Methionine 671 of Human AβPP

Below, we describe four distinct mechanisms potentially capable of generating human *i*Aβ independently of AβPP. All these mechanisms have one common attribute: in each mechanism under discussion translation of AβPP initiates from the AUG codon for Met671 of human AβPP [1,2,3,4,5,6,7]. The pivotal role of this codon is a direct consequence of its singular location within human AβPP mRNA. Human AβPP cDNA was cloned and sequenced by several research groups in 1987 [126,127,128]. Following publication of its nucleotide sequence, another research group noticed that the segment encoding the C99 fragment of AβPP is preceded contiguously and in-frame by the AUG codon (normally encoding Met671 of AβPP) [129]. Moreover, this AUG codon is situated within the optimal translation initiation nucleotide context [129]. Furthermore, the singularity of this codon is reflected in the observation that, of 20 methionine-encoding codons in human AβPP mRNA, the AUG encoding Met671 of AβPP is the only one situated within the optimal translation initiation nucleotide context; remarkably, not even the AUG initiating translation of human AβPP (i.e., encoding its Met1) is located within the optimal nucleotide context [129]. The singularity of the AUG codon in question implied that it reflects the physiological function, i.e., that this codon can potentially serve in the initiation of translation from this position and, were this the case, such translation would result in C99 and, subsequently, in Aβ generated independently of AβPP.

#### 4.2.2. Molecular Mechanisms Potentially Capable of Generating *i*Aβ Independently of AβPP

Accordingly, the authors of [129] suggested that the physiological function reflected in the singular position of the AUG encoding Met671 is the internal initiation of translation of the intact human AβPP mRNA [129]. Following this proposal, two research groups attempted to test it [130,131]. The rationale in both cases was similar: if translation initiates internally, from the AUG codon in question, changes within the coding region upstream from this AUG would not impede it. In one study, various frame-shifting mutations were introduced upstream from the AUG encoding Met671 of AβPP, whereas in another study, translational stop codon was implanted upstream from this AUG. In both cases, it was expected that if the internal initiation of translation occurs, the manipulations described above would not interfere with it. In both cases, however, manipulations prevented production of C99 and Aβ; both groups concluded that the internal initiation of translation of the intact AβPP mRNA could be ruled out [130,131]. This conclusion, however, was not justified. The internal initiation of the translation was proposed in [129] to take place in AD-affected human neuronal cells, but the tests were performed not in the neuronal cells, and certainly not under the AD conditions. Therefore, the proposed internal initiation of translation of the intact human AβPP mRNA from the AUG encoding Met671 [129] remains viable and should be re-assessed in an appropriate model system (discussed in [7]).

In the above scenario, it was proposed [129] that C99 could be generated independently of AβPP by the unconventional internal initiation of translation of the intact AβPP mRNA from the AUG normally encoding Met671. In the following three scenarios, however, the same outcome can be accomplished conventionally via translation of human AβPP mRNA that is 5′-truncated in such a way that the AUG normally encoding methionine residue 671 becomes the first functional translation initiation codon [4,7]. In one such scenario [4,7], the appropriately 5′-truncated human AβPP mRNA is generated by the internal initiation of transcription of the AβPP gene. This would necessitate expression in the AD-affected neuronal cells of a specialized transcription factor, which would presumably occur following the ISR-triggered transcriptional and translational reprogramming.

In another scenario [4,7], an appropriately 5′-truncated AβPP mRNA is generated by the site-specific cleavage within the intact AβPP mRNA upstream from the AUG encoding Met671. This would require expression of a specialized nuclease occurring, presumably, as a consequence of the ISR-triggered transcriptional and translational reprogramming. The truncated AβPP mRNA product would be distinct from that generated by the internal initiation of transcription because it would lack the 5′-terminal cap”G”. It would, nevertheless, be preferentially translated under the ISR conditions. 

In the final scenario, the suitably 5′-truncated human AβPP mRNA is generated as the outcome of the chimeric RNA-dependent asymmetric amplification of AβPP mRNA [132,133,134,135,136,137,138,139,140,141,142] (reviewed in [4,7]). It is “chimeric” because its 5′ untranslated region (5′UTR) contains a 3′-terminal segment of the antisense AβPP mRNA, and it is “asymmetric” because only the 3′ portion of AβPP mRNA is amplified and it encodes only the C100 fragment of human AβPP. The first 5′-most translation initiation codon in the resulting chimeric mRNA is, in fact, the AUG normally encoding the Met671 of AβPP. Arguably, this scenario is the most plausible one because, unlike the remaining scenarios, it is strongly supported by the empirical data [143,144,145,146,147]. In this scenario, reviewed in [7], every conventionally genome-transcribed human AβPP mRNA serves repeatedly as an amplification template, the equivalent of a massive gene amplification. Consequently, its *i*Aβ-generating efficiency would, presumably, be orders of magnitude greater than that of the AβPP proteolysis. This scenario also provides a mechanistic explanation as to why Alzheimer’s disease occurs in humans but not in animals and in the current transgenic animal models of AD [7].

#### 4.2.3. The Primary Translation Product of the AβPP-Independent *i*Aβ Production Pathway Is C100 (N-Terminal Met-C99) Rather than C99: Experimental and Diagnostic Implications

At the time of publication of [129], it was assumed that C99 and Aβ produced independently of AβPP via the initiation of the translation from the AUG normally encoding Met671 of AβPP would be indistinguishable from those generated by AβPP proteolysis because the initiating methionine would be removed co-translationally by N-terminal methionine aminopeptidase (MAP). As transpired in subsequent investigations of the N-terminal methionine processing, however, this turned out not to be the case. 

Methionine initiates translation of the decisive bulk of cellular proteins. It is usually removed concurrently with translation by MAP1 or MAP2, and in the primary translation product, the N-terminal residue is the one that follows the translation-initiating methionine. This, as mentioned above, turned out not to be always the case [148,149,150,151,152,153]. Cleavage of the N-terminal methionine takes place within the active site of MAP1/MAP2, and for it to occur, both N-terminal Met and the following residue must be accommodated within this site; the feasibility of such accommodation depends on the size of the second residue (since size of Met is constant) and it is not always feasible. Only the seven smallest amino acid residues can be accommodated within the active site of MAP1/MAP2 together with Met. They are: glycine, serine, cysteine, threonine, proline, and valine [148,149,150,151,152]. No amino acid residue larger than valine can be accommodated together with N-terminal methionine within the active site of MAP1/MAP2, and when such combination occurs, the translation-initiating Met is not cleaved off co-translationally and is retained in the primary translation product [148,149,150,151,152,153]. 

Methionine residue 671 of AβPP is followed by the aspartate, which is much larger than valine. The N-terminal Met/Asp pair cannot be accommodated within the active site of MAP. Consequently, if, regardless of the underlying mechanism, translation of the intact or 5′-truncated human AβPP mRNA initiates with Met671, it would not be removed co-translationally. The primary product of such translation would therefore be not C99, but rather C100, i.e., N-terminal Met-C99. In such cases, when the translation-initiating methionine is not removed co-translationally by MAP1/MAP2, it is typically cleaved off by one of aminopeptidases with a broad specificity [153]. Therefore, C100 would be subsequently (post-translationally) converted to C99. Provided that C100 undergoes the gamma-cleavage prior to the removal of the translation-initiating methionine, N-terminal Met-Aβ would result, and would subsequently be converted into Aβ by the same process that converts C100 into C99.

Crucially, the cleavage of the translation-initiating Met by an aminopeptidase other than MAP1 or MAP2 invariably occurs post- rather than co-translationally. This implies that cellular pools of C100 and N-terminal Met-Aβ should occur in AD-affected human neurons [1,2,3,4,5,6,7]. Sizes of these pools would depend on the rate of cleavage of the N-terminal Met, and, for Met-Aβ, on the ratio of the rate of aminopeptidase cleavage to that of gamma-secretase cleavage. In any case, these pools should be present in live AD-affected neuronal cells, and their occurrence would report on the activity of the AβPP-independent *i*Aβ generation pathway. It should be emphasized that such pools cannot occur in the postmortem samples because, in dying cells, proteolysis continues long after protein synthesis ceases, and in the absence of C100 influx, the C100 to C99 and Met-Aβ to Aβ conversions would be complete.

## 5. Dynamics of *i*Aβ in Conventional Alzheimer’s Disease

### 5.1. Dynamics of iAβ Accumulation in Health and AD

**Figure 1:**
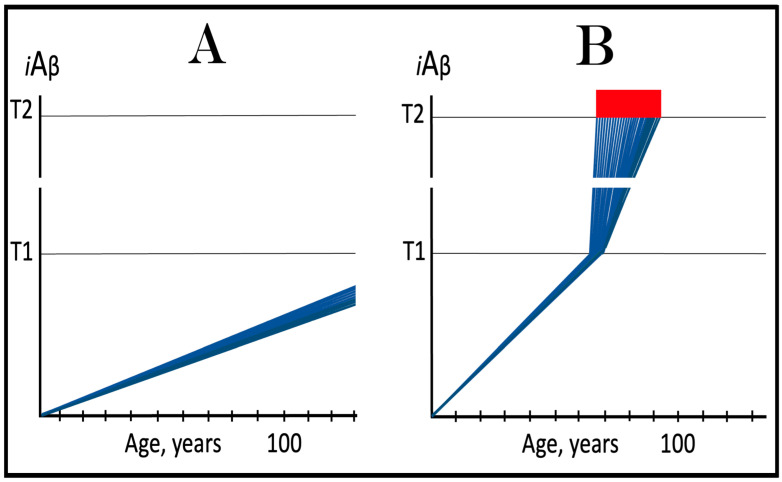
**Dynamics of *i*Aβ accumulation in conventional AD.** *i*Aβ: Intraneuronal Aβ. ***T1*** *threshold*: Cellular concentration of *i*Aβ that triggers, via eIF2α kinases, and the integrated stress response, activation of the AβPP-independent *i*Aβ generation pathway. ***T2*** *threshold*: Cellular concentration of *i*Aβ, produced predominantly in the AβPP-independent pathway, which triggers apoptosis or necroptosis of neuronal cells. *Blue lines*: Levels of *i*Aβ in individual neurons. *Red Box*: Apoptotic Zone, the continuum of *i*Aβ levels that lead irreversibly to cell death. *Panel* (**A**): AβPP-derived *i*Aβ does not reach the T1 threshold within the lifespan of an individual; no AD occurs. *Panel* (**B**): AβPP-derived *i*Aβ crosses the T1 threshold; PKR and/or HRI kinases are activated, eIF2α is phosphorylated, the integrated stress response is elicited, the autonomous AβPP-independent *i*Aβ generation pathway is initiated, and AD commences and progresses. For details, see the main text.

In the ACH2.0 perspective, healthy individuals, i.e., persons who do not develop conventional AD within their lifetimes, remain healthy because, in their neurons, AβPP-derived *i*Aβ does not reach the “critical threshold”. This scenario, which is apparently prevailing in the general population, is illustrated diagrammatically in Figure 1A. The dynamics of *i*Aβ accumulation is single-phased and linear. Aβ is produced exclusively by the AβPP proteolysis, and accumulation of *i*Aβ occurs throughout the lifetime via its importation from the extracellular pool and intraneuronal retention of a fraction generated by C99 cleavage within cellular organelles. The levels of AβPP-derived *i*Aβ do not reach the T1 threshold (the “critical threshold”). Consequently, PKR and/or HRI kinases are not activated, eIF2α is not phosphorylated, the IRS is not elicited, the AβPP-independent *i*Aβ generation pathway is not initiated, and AD does not occur. As was mentioned above, the T1 threshold, i.e., the levels of AβPP-derived *i*Aβ required for the activation of PKR could be distinct from that needed for the activation of HRI. This, however, is inconsequential, since the activation of either is sufficient to elicit the ISR and therefore the “T1 threshold” is used as a generic designation.

In contrast to the single-phased kinetics of *i*Aβ accumulation in healthy individuals, the dynamics of the accrual of *i*Aβ in AD patients, shown in Figure 1B, is two-phased. In the first phase, only *i*Aβ derived by the proteolysis of AβPP accumulates, and the only distinction between healthy persons and future AD patients is either the rate of the accrual of AβPP-derived *i*Aβ, or the extent of the T1 threshold. In AD patients, the former is faster, and the latter is potentially lower than in healthy individuals. Consequently, the T1 threshold is reached and crossed by AβPP-derived *i*Aβ within the lifespan of an AD patient. This signifies the transition to the second AD phase. PKR and/or HRI kinases are activated, eIF2α is phosphorylated, the ISR is elicited, the AβPP-independent *i*Aβ production pathway becomes operational, and AD commences. The levels of *i*Aβ, produced in this phase overwhelmingly predominantly in the AβPP-independent pathway, rapidly increase and drive the AD pathology [154,155,156,157]. When these levels reach the T2 threshold and cross into the “Apoptotic Zone” (red box), apoptosis or necroptosis [158] of the affected neuronal cells ensue. 

### 5.2. Conventional AD Is a Two-Stage Disease, but Stage One Is Conditional

Two phases of the accrual of *i*Aβ correspond to two stages of the disease. The first stage is asymptomatic. In fact, there is no disease at this stage. AD commences, and its symptoms manifest only at the second stage. At this point, as discussed below, the interference with the dynamics of the accrual of AβPP-derived *i*Aβ would be futile because AD is powered by *i*Aβ generated independently of AβPP [1,2,3,4,5,6,7]. Describing the accrual of AβPP-derived *i*Aβ preceding the crossing of the T1 threshold as the first stage of AD is paradoxical. As mentioned above, there is evidently no AD at this stage. And unless the levels of AβPP-derived *i*Aβ do cross the T1 threshold during the lifetime of a person, no AD would occur and “the first stage of AD” would be an oxymoron. This is exactly what takes place in the majority of the population, and is depicted in Figure 1A. Nevertheless, it is a useful term, provided it is applied conditionally. In other words, “the first stage of AD” becomes indeed the first stage of AD only post-factum, if/when the T1 threshold is reached, the ISR elicited, the AβPP-independent *i*Aβ generation pathway initiated, and the disease occurs. In any other case, the life-long accrual of AβPP-derived *i*Aβ to levels below the T1 threshold would be just a normal physiological (and inconsequential, i.e., non-deleterious) process.

### 5.3. All Known AD-Causative and AD-Preventive Mutations Act by Altering the Dynamics of iAβ Accumulation

The preceding account of the kinetics of the accrual of AβPP-derived *i*Aβ implies that it defines the occurrence of AD, as well as its timing. The greater the rate of the accrual of AβPP-derived *i*Aβ is, and the smaller the value of the T1 threshold, the sooner would the latter be crossed and the disease would occur [4,7]. Conversely, the lesser the rate of the accrual of AβPP-derived *i*Aβ is, and the greater the T1 threshold, the later would the T1 threshold be reached and the disease would commence. And provided the T1 threshold is not crossed during the lifetime of a person, no AD would occur [4,7]. The above statements fairly accurately describe the mechanistic effects of all known mutations and factors that predispose to conventional AD, cause it, or protect from it. As an example, the cellular uptake of secreted Aβ involves ApoE, which occurs in several isoforms, with ApoE4 far more efficient in importation of Aβ than others [32]. Because of this, it increases the rate of accumulation of AβPP-derived *i*Aβ and predisposes to the disease [32]. In another instance, certain mutations of presenilins significantly increase the proportion of gamma-cleavages at the position 42 of Aβ [51] and, consequently, secretion of this Aβ isoform, which, in turn, is imported by the cell with the efficiency twice that of other isoforms of Aβ [50]. In shifting gamma-cleavage to the position 42 of Aβ, these mutations elevate the rate of accrual of AβPP-derived *i*Aβ and thus cause the early onset of the disease [51]. The Swedish Aβ mutation [159] and certain presenilins mutations [160] increase the proportion of gamma cleavages occurring on the intracellular rather than on plasma membranes [159,160]. This causes the increased retention of AβPP-derived *i*Aβ, elevates its rate of accumulation, and causes familial AD. Cleavages within *i*Aβ are known to occur physiologically and to limit both production of Aβ and accumulation of *i*Aβ. The Flemish Aβ mutation reduces the rate of some of these cleavages [161]. This elevates the rate of accrual of AβPP-derived *i*Aβ, and also causes familial AD. In contrast, the Icelandic Aβ mutation increases the rate of physiologically occurring cleavages within *i*Aβ. This decreases the rate of the accrual of AβPP-derived *i*Aβ and protects from AD as well as from aging-associated cognitive decline, AACD [25,26].

### 5.4. Given Sufficient Longevity, Conventional AD Is Inevitable

It follows from the above that individuals who do not develop conventional AD during their lifespans (the vast majority of the general population), including carriers of the protective Icelandic mutation, are resistant to the disease due solely to the low rate of accrual of AβPP-derived *i*Aβ. If the T1 threshold is not reached during their lifetimes, no conventional AD occurs. “During their lifetimes” is quintessential in the preceding statement. This is because in individuals who do not develop AD, it is the limited lifespan, which makes them “resistant” to the disease; they simply run out of time, the lifetime. If there were no limitation on the duration of individuals’ lifespans, the current situation where the majority of the population does not develop AD would be reversed diametrically: every individual would inevitably develop the disease, provided their lifespans are long enough. Since the accrual of AβPP-derived *i*Aβ is a lifelong process, it is just a function of time when the T1 crossing will occur, but occur it will given sufficient time, and the disease will ensue. While this consideration may appear purely hypothetical, in reality it is not. The longevity of the general population is steadily increasing, and the proportion that develops the disease increases accordingly. With this trend continuing, a probability of developing the disease might become the eventuality unless a preventive therapy is available [1,2,3,4,5,6,7].

## 6. The AD Engine: *i*Aβ Generated Independently of AβPP Drives the AD Pathology; It Also Propagates the Neuronal ISR and Thus Perpetuates Operation of the AβPP-Independent Pathway of Its Own Production

To summarize the preceding discussion, the ACH2.0 defines conventional Alzheimer’s disease as a two-stage process. In stage one, AβPP-derived *i*Aβ accrues physiologically through two distinct pathways. One is the retention of *i*Aβ generated by the gamma cleavage of C99 within cellular organelles. Another pathway is the importation of *i*Aβ from the extracellular Aβ pool. These two pathways operate physiologically in both healthy individuals and future AD patients, and the outcome is decided by the dynamics of AβPP-derived *i*Aβ accumulation. The AβPP-derived *i*Aβ accrual process becomes the first stage of AD only if/when it crosses the T1 threshold and triggers the second stage of the disease. In stage two of AD, sufficient levels of AβPP-derived *i*Aβ mediate activation of PKR and/or HRI kinases. One or both of these kinases phosphorylate, in a site-specific manner, eIF2α, and thus elicit the integrated stress response in neuronal cells. Within the framework of the ISR, both transcription and translation undergo a substantial reprogramming. As the result, the cap-dependent translation is inhibited and total protein synthesis is severely suppressed. This is accompanied by a simultaneous activation of production, apparently in a cap-independent mode, of a subset of cellular proteins. The latter apparently includes essential components of the AβPP-independent *i*Aβ generation pathway, which are necessary for its operation and are “missing” under regular conditions. 

**Figure 2:**
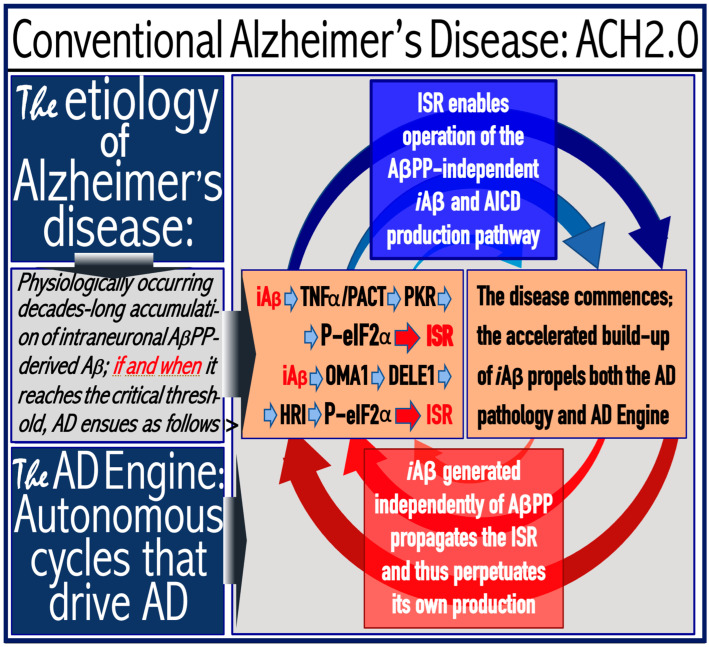
**Etiology of conventional Alzheimer’s disease: The ACH2.0 perspective.** *i*Aβ: Intraneuronal Aβ. *eIF2α*: Eukaryotic translation initiation factor 2alpha. *PKR*, *HRI*: Kinases that phosphorylate eIF2α. *TNFα*: Tumor necrosis factor alpha (presumably activates PKR). *PACT*: PKR activator. *OMA1*: Mitochondrial protease activated by mitochondrial dysfunction. *DELE1*: Mitochondrial protein mediating, following its cleavage by OMA1, activation of HRI. *ISR*: Integrated stress response; it is elicited by phosphorylation of eIF2α and, in turn, activates the AβPP-independent *i*Aβ production pathway. *AICD*: AβPP intracellular domain. *Grey Box (left)*: Physiologically occurring intraneuronal accumulation of *i*Aβ. Provided *i*Aβ crosses the critical threshold, it triggers activation of PKR and/or HRI. *Pink Box (left)*: Phosphorylation of eIF2α and elicitation of the integrated stress response lead to radical transcriptional and translational reprogramming, resulting in the production of components required for the operation of the AβPP-independent *i*Aβ generation pathway. *Blue Box*: With necessary components provided, the AβPP-independent *i*Aβ generation pathway is activated; its end products, *i*Aβ and AICD, are retained within the neurons. *Pink Box (right)*: Levels of *i*Aβ, generated in the AβPP-independent *i*Aβ production pathway, rapidly increase and drive the AD pathology. *Red Box*: High levels of *i*Aβ sustain the activity of eIF2α kinases, propagate the ISR conditions, and perpetuate the operation of the AβPP-independent pathway of its own production. The repeated cycles denoted by arched arrows constitute an engine that drives AD, the AD Engine. Note that the present image was used in [7].

The availability of its crucial components activates the AβPP-independent *i*Aβ production pathway; this signifies the commencement of AD. Since the entire *i*Aβ output of this pathway is retained intraneuronally, its cellular concentration increases rapidly, resulting in two major corollaries. One is that *i*Aβ generated independently of AβPP attains (in contrast to its counterpart produced by the AβPP proteolysis) levels sufficient to drive the AD pathology, leading to formation of NFTs [154,155,156,157] and neuronal death. Another major consequence is that the elevated levels of *i*Aβ support its own production in the AβPP-independent pathway. Indeed, sufficient levels of *i*Aβ sustain the activity of PKR and/or HRI kinases, thus maintaining eIF2α in the phosphorylated state and consequently propagating the ISR and perpetuating the operation of the AβPP-independent pathway of its own generation. The continuous cycles of *i*Aβ-stimulated propagation of its own production constitute an engine that propels AD, the “AD Engine”; its origins and the mode of operation are illustrated diagrammatically in Figure 2. 

In conventional AD, once the AβPP-independent *i*Aβ generation pathway is activated, it is immediately self-sustainable, and the AD Engine becomes operational. This is because, at this stage, the levels of *i*Aβ (AβPP-derived) have crossed the T1 threshold. This point seems trivial and self-evident in terms of the ACH2.0, but it attains significance in the following sections where unconventional classes of AD are discussed. It should be also noted that concurrently with the increase in levels of *i*Aβ, the AβPP-independent pathway elevates the levels of AβPP intracellular domain, AICD that was shown to be capable of interference with various components of AD [162,163,164,165,166,167,168,169,170,171,172,173,174,175,176,177] but whose potential involvement in the disease remains to be fully elucidated (reviewed in [4,7]).

## 7. Unconventional Alzheimer’s Disease: Elicitation of the Neuronal ISR by Stressors Other Than AβPP-Derived *i*Aβ

### 7.1. Operational Definition of AD

It follows from the above discourse that AD is a disease driven by *i*Aβ generated in the self-sustainable and autonomous AβPP-independent pathway. It commences with the activation of this pathway, and, on the cellular level, progression of the disease parallels and reflects accumulation of *i*Aβ; when the latter reaches the T2 threshold, neurons commit apoptosis or necroptosis. All processes leading to activation of the AβPP-independent pathway are either harmless/asymptomatic or reversible, or both. Indeed, the reversal of any of those processes, including the elicitation of the ISR, would preclude the disease. On the other hand, as soon as the AβPP-independent *i*Aβ generation pathway is self-sustainably operative, i.e., as soon as the AD Engine is active, the disease is unstoppable and irreversible (unless interfered with therapeutically) [1,2,3,4,5,6,7]. This definition of AD encompasses both the conventional and unconventional forms of the disease; the only distinction between the two is the manner of their causation.

### 7.2. Following the Logic of the ACH2.0: Another, Unconventional Class of AD Has to Occur

In the ACH2.0 paradigm, the disease is caused by *i*Aβ produced in the AβPP proteolytic pathway and accumulated, in a decades-long process, to sufficient levels [1,2,3,4,5,6,7]. When it crosses the T1 threshold, PKR and/or HRI kinases are activated, eIF2α is phosphorylated at the serine residue 51, and the integrated stress response is elicited in neuronal cells. The elicitation of the ISR is the pivotal point, the last reversible event in the development of AD. As soon as the components required for operation of the AβPP-independent *i*Aβ generation pathway are produced, within the ISR framework, the pathway is activated; it is autonomous and irreversible, and thus the disease, which it propels, is irrevocable physiologically. If we pursue this logic and follow the above definition of the disease, it becomes apparent that another, unconventional, class of Alzheimer’s disease has to occur. This is because the integrated stress response in neuronal cells can be elicited by stressors distinct from AβPP-derived *i*Aβ.

### 7.3. Operational Definition of Unconventional Neuronal ISR-Initiated AD

**Figure 3:**
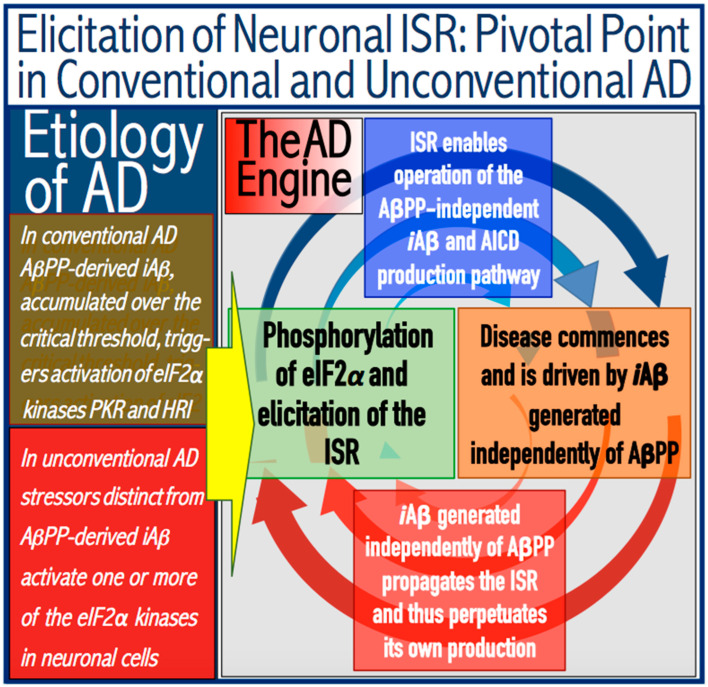
**Elicitation of neuronal integrated stress response is the pivotal point in both conventional and unconventional ISR-initiated Alzheimer’s disease.** *i*Aβ: Intraneuronal Aβ. *eIF2α*: Eukaryotic translation initiation factor 2alpha. *PKR*, *HRI*: Kinases that phosphorylate eIF2α. *ISR*: Integrated stress response; it is elicited by phosphorylation of eIF2α and, in turn, activates the AβPP-independent *i*Aβ production pathway. *AICD*: AβPP intracellular domain. In conventional AD, the stressor that triggers activation of PKR and HRI kinases is AβPP-derived *i*Aβ accumulated over the T1 threshold. In unconventional AD, activation of one or more of the eIF2a kinases is triggered by stressors other than AβPP-derived *i*Aβ. The distinct pathways leading to conventional and unconventional forms of AD merge mechanistically at the stage of phosphorylation of eIF2α. This event leads to the pivotal point in both forms of AD, namely the elicitation of the ISR, which enables production of the essential components of, and thus activates the, AβPP-independent *i*Aβ generation pathway. The *i*Aβ product of this pathway reaches levels sufficient to drive the AD pathology. It also sustains the activity of eIF2α kinases, propagates the ISR conditions, and perpetuates the operation of the AβPP-independent pathway of its own production.

Indeed, the elicitation of the ISR can potentially occur in a multitude of ways, by numerous stressors distinct from AβPP-derived *i*Aβ and capable of activating any one of the four eIF2α kinases. There is no reason to presume that this does not occur in neuronal cells; to the contrary, there is every reason to believe that it does. In such a case, subsequent elicitation of the ISR would provide the necessary components for and activate the AβPP-independent *i*Aβ generation pathway. If operation of this pathway becomes self-sustainable and autonomous (discussed in more detail below), unconventional AD would ensue. From this point on, the disease would be mechanistically identical to conventional AD. Its unconventionality, i.e., its distinction from conventional AD, would be only in the sequence of causative events leading to elicitation of the ISR and activation of the self-sustainable AβPP-independent *i*Aβ production pathway. *Therefore, this class of unconventional AD can be operationally defined as Alzheimer’s disease triggered by the neuronal integrated stress response elicited by a stressor distinct from AβPP-derived iAβ. Moreover, unconventional AD always initiates at the levels of AβPP-derived iAβ below the T1 threshold because the T1 crossing triggers the conventional form of the disease*. This is in contrast to conventional AD, which is defined as the disease initiated by AβPP-derived *i*Aβ accumulated over the T1 threshold. The notion that both conventional and unconventional forms of AD are, from the instance of their commencement, mechanistically identical (i.e., driven by *i*Aβ generated in the self-sustainable AβPP-independent pathway), and both are induced by the neuronal ISR elicited differentially via distinctly diverse stressors, is illustrated in Figure 3.

### 7.4. Conditions Associated with and Potentially Causing Unconventional ISR-Related AD

Conditions that are associated with unconventional AD, which potentially cause the disease by triggering elicitation of the sustained integrated stress response in neuronal cells, are numerous. They include traumatic brain injury (TBI) and chronic traumatic encephalopathy (CTE) [178,179,180,181]. They also include viral [182,183,184,185,186] and bacterial [187,188,189,190] infections. Indeed, it has been shown that a number of herpes viruses, such as HHV1, HHV2, HHV3 (varicella zoster virus), HHV4 (Epstein-Barr virus), HHV5 (cytomegalovirus), HHV6A, HHV6B and HHV7, flaviviruses, such as Zika virus, Dengue fever virus, and Japanese encephalitis virus, human immunodeficiency virus, HIV, hepatitis viruses, such as HAV, HBV, HCV, HDV, HEV, SARS-CoV2 virus, Ljungan virus, influenza A virus, and Borna disease virus are associated with, increase the risk of, and can potentially cause AD. As an example, a person with viral encephalitis is thirty times as likely to be diagnosed with AD as someone without encephalitis [182,183,184,185,186]. A similar link was found between bacterial infections and AD. Thus, infection with spirochetal bacteria is associated with a ten-fold increased occurrence of AD, and a five-fold increase in in the occurrence of AD was caused by infection with Chlamidophyla pneumonia [187,188,189,190]. Neuroinflammation, systemic inflammation in general, and such inflammatory conditions as osteoarthritis and rheumatoid arthritis in particular, have been also strongly associated with Alzheimer’s disease [191,192,193,194,195,196,197,198,199,200,201,202].

### 7.5. The “Duck Test”: If It Looks Like a Duck, Walks Like a Duck, Quacks Like a Duck, Maybe It Is a Duck?

It has long been recognized that certain maladies listed above are not only associayed withAD but cause conditions referred to as “AD-like dementia” (ADLD) or “AD-related dementia” (ADRD). A typical case in this respect is that of chronic traumatic encephalopathy (and of TBI to a certain degree). Both AD and CTE (or rather the condition caused by the latter) are characterized by similar cognitive impairments. They also share the key neuropathological features, most notably neurofibrillary tau tangles, the major, actually ultimate, hallmark of the disease [178,179,180,181]. Is the condition caused by CTE actually Alzheimer’s disease? An assessment referred to as the “duck test” is applicable in this situation. The test is formulated as follows: if it looks like a duck, walks like a duck, quacks like a duck, it is probably a duck. The CTE case clearly passes the duck test. The condition caused by it is probably unconventional Alzheimer’s disease masked by the absence of excessive deposition of extracellular Aβ (because it is unconventional AD and thus is not associated with the overproduction of AβPP-derived Aβ) and by the overlapping condition resulting from brain injuries. This consideration is probably applicable to many, if not most, cases of ADLD and ADRD that can turn out, in fact, to be the cases of unconventional AD.

### 7.6. Pathways That Are Common to the AD-Associated Conditions and Can Result in Elicitation of the ISR in Neuronal Cells

The question is: how do the various conditions discussed above transduce their signals to the neuronal cells and consequently cause AD? The answer is: probably in as various and numerous ways as the conditions themselves occur. But there are also, apparently, signal transduction features common to most, possibly all, of the above conditions. One such common feature is the impairment of the blood–brain barrier (BBB). Indeed, many of the AD-associated, possibly AD-causing conditions discussed above, such as TBI, CTE, persistent infections, and systemic inflammation, impair and compromise the BBB [203]. The compromised BBB, in turn, opens up plentiful opportunities for the penetration of stressors into the brain in general and the neurons in particular. An even more universal feature that is common to all conditions potentially causing Alzheimer’s disease is the reduction in cerebral blood flow (CBF) [204,205,206,207,208]. The association of the reduced CBF with AD is, in fact, so strong that a theory has been advanced, decades ago, naming the reduced CBF as the primary cause of AD [209,210]. The reduction in CBF can potentially affect neurons in many ways. Of great interest is the established notion that a reduction in the cerebral blood flow causes mitochondrial dysfunction in the neuronal cells [211,212]. As discussed above, mitochondrial distress would result in the activation of mitochondrial protease OMA1, cleavage of another mitochondrial protein, DELE1, exportation of one of the resulting DELE1 fragments to the cytosol, and its binding to and consequent activation of the HRI kinase [89,90]. With the activation of HRI, phosphorylation of eIF2α, elicitation of the neuronal integrated stress response, activation of the AβPP-independent *i*Aβ generation pathway, and commencement of unconventional Alzheimer’s disease would follow. The occurrence of this pathway illustrates how a signal from a distant event such as, for example, osteoarthritis of the knee can, via systemic inflammation-associated signal transduction, reach neurons in the brain and trigger unconventional AD.

## 8. Pursuing the Logic Further: Unconventional Alzheimer’s Disease Initiated via ISR-Unrelated Activation of the AβPP-Independent *i*Aβ Generation Pathway

The preceding section introduced an unconventional class of Alzheimer’s disease where the commonality with conventional AD begins at the stage of elicitation of the neuronal ISR, which is, in contrast to conventional AD, effected by stressors other than AβPP-derived *i*Aβ. In both, conventional and unconventional AD, the very same mechanism, namely the AβPP-independent generation of *i*Aβ, drives the AD pathology, and in both this active core is induced by elicitation of the neuronal integrated stress response, which provides essential components necessary for operation of the AβPP-independent *i*Aβ production pathway. The difference between this unconventional and conventional forms of AD is solely in the way the eIF2α is phosphorylated and the ISR elicited: in the conventional disease, the stressor activating the eIF2α kinases is AβPP-derived *i*Aβ accumulated, in a decades-long process, over the critical threshold, whereas in unconventional AD eIF2α, kinases are activated and, consequently, the ISR elicited by stressors other than AβPP-derived *i*Aβ.

**Figure 4:**
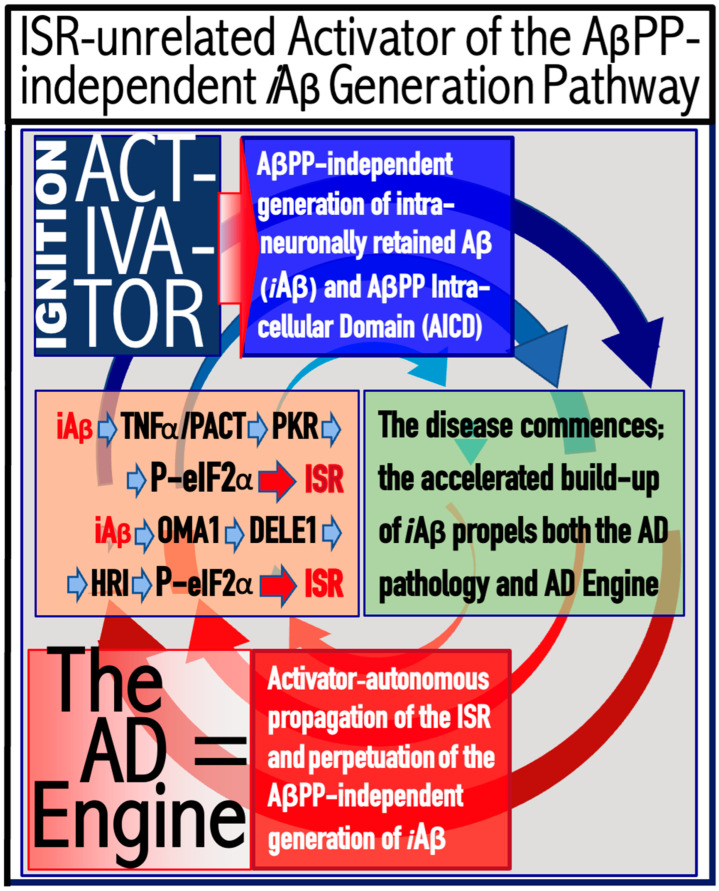
**Unconventional ISR-independent activation of the AβPP-independent *i*Aβ generation pathway: another potential class of unconventional AD.** *i*Aβ: Intraneuronal Aβ. *eIF2α*: Eukaryotic translation initiation factor 2alpha. *PKR*, *HRI*: Kinases that phosphorylate eIF2α. *TNFα*: Tumor necrosis factor alpha (presumably activates PKR). *PACT*: PKR activator. *OMA1*: Mitochondrial protease activated by mitochondrial dysfunction. *DELE1*: Mitochondrial protein mediating, following its cleavage by OMA1, activation of HRI. *ISR*: Integrated stress response; it is elicited by phosphorylation of eIF2α and, in turn, activates the AβPP-independent *i*Aβ production pathway. *AICD*: AβPP intracellular domain. *Activator*: A substance capable of activating the AβPP-independent *i*Aβ generation pathway independently of the ISR. It initiates and maintains operation of the AβPP-independent *i*Aβ generation pathway in the absence of the ISR. When *i*Aβ produced in this pathway accumulates over the T1 threshold, it triggers activation of PKR and/or HRI kinases and elicitation of the integrated stress response. From this point on, the ISR sustains operation of the AβPP-independent *i*Aβ generation pathway autonomously from the initial activator. *i*Aβ, continuously generated independently of AβPP, is maintained above the T1 threshold. It propagates the ISR and thus perpetuates its own production; the AD Engine is operational, and the disease commences.

The logic of this approach, i.e., ignition of the AD Engine in ways distinct from the accumulation of AβPP-derived *i*Aβ over the T1 threshold, can be pursued even further. Indeed, besides the elicitation of the ISR in AβPP-derived *i*Aβ-independent manner, there is one more opening to accomplish the initiation of the physiologically irreversible, self-sustained operation of the AD Engine: the direct, ISR-unrelated, activation of the AβPP-independent *i*Aβ generation pathway. This process is illustrated in Figure 4. 

In this figure, an activator initiates and maintains operation of the AβPP-independent *i*Aβ generation pathway in the absence of the ISR. When *i*Aβ produced in this pathway accumulates over the T1 threshold, it triggers activation of PKR and/or HRI kinases and elicitation of the integrated stress response. From this point on, the ISR sustains operation of the AβPP-independent *i*Aβ generation pathway autonomously and independently from the initial activator. *i*Aβ, continuously generated independently of AβPP, propagates the ISR and thus perpetuates its own production; the AD Engine becomes operational, and the disease commences. The withdrawal of the initial activator would be inconsequential at this stage. AD induced by such an activator would constitute the second class of unconventional Alzheimer’s disease. As for the nature of this putative activator, it could be a “missing” component of the AβPP-independent *i*Aβ generation pathway (provided by the ISR in conventional AD and in the first class of unconventional AD) produced independently of the ISR (the feasibility of this pathway is further discussed in Section 9.4 below).

## 9. ACH2.0/E: The Consolidated Theory of Conventional and Unconventional Alzheimer’s Disease

### 9.1. Conventional and Unconventional Forms of AD Are Driven by the Same Common Mechanism; the Distinction Is Only in the Manner of Its Activation

Above, three classes of Alzheimer’s disease have been considered. In each class, the disease commences with the initiation of self-sustainable operation of the AβPP-independent *i*Aβ generation pathway. In each class, this pathway is the active core of the disease; it drives the AD pathology, and propagates (by maintaining the ISR state) and perpetuates its own activity. In this respect, there is no distinction between different classes of AD. What defines them as the distinct entities is the manner in which autonomous operation of the AβPP-independent *i*Aβ production pathway is attained. In conventional AD, the stressor that triggers activation of the AβPP-independent pathway of *i*Aβ production is AβPP-derived *i*Aβ accumulated over the critical threshold. It mediates activation of the PKR and/or HRi kinases, which, in turn, phosphorylate eIF2α thus eliciting the integrated stress response. The ISR severely suppresses the total cellular protein production, but simultaneously activates the synthesis of a small subset of cellular proteins. Among them, presumably, are components necessary for operation of the AβPP-independent *i*Aβ generation pathway; when they are available, the pathway is activated. In one of the unconventional AD classes, the identical sequence of events takes place, with one exception: an eIF2α kinase (any one of the four) is activated by a stressor other than AβPP-derived *i*Aβ. In the second class of unconventional AD the initial activation of the AβPP-independent *i*Aβ generation pathway occurs independently of the ISR. 

### 9.2. ACH2.0/E: Consolidated Interpretation of Conventional and Unconventional Alzheimer’s Disease

**Figure 5:**
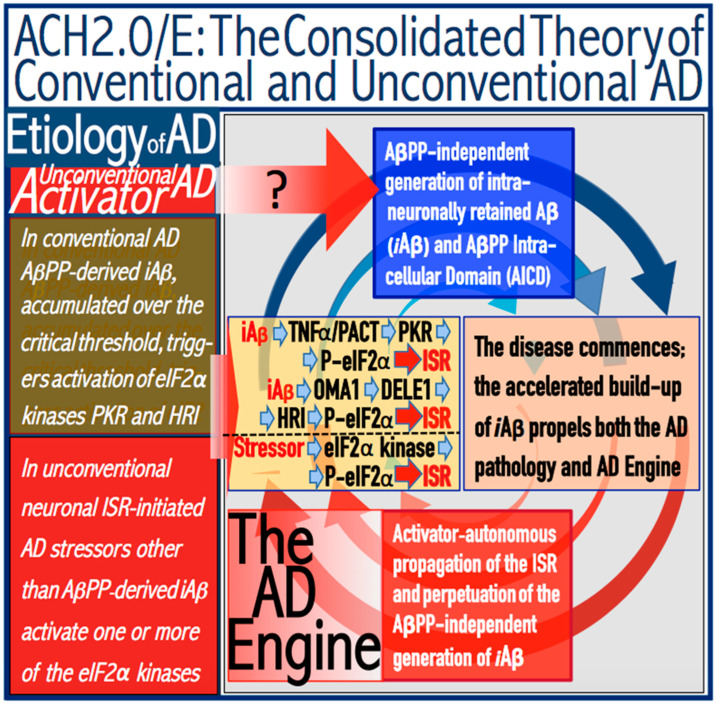
**ACH2.0/E: The consolidated interpretation of conventional and unconventional Alzheimer’s disease.** *i*Aβ: Intraneuronal Aβ. *eIF2α*: Eukaryotic translation initiation factor 2alpha. *PKR*, *HRI*: Kinases that phosphorylate eIF2α. *TNFα*: Tumor necrosis factor alpha (presumably activates PKR). *PACT*: PKR activator. *OMA1*: Mitochondrial protease activated by mitochondrial dysfunction. *DELE1*: Mitochondrial protein mediating, following its cleavage by OMA1, activation of HRI. *ISR*: Integrated stress response; it is elicited by phosphorylation of eIF2α and, in turn, activates the AβPP-independent *i*Aβ production pathway. *AICD*: AβPP intracellular domain. *Activator*: A substance capable of activating the AβPP-independent *i*Aβ generation pathway independently of the ISR. *Stressor*: A substance, which is capable of activating one or more of the eIF2α kinases and is distinct from AβPP-derived *i*Aβ. *Question mark*: Feasibility of this pathway is conditional; see Section 9.4 of the main text. This figure illustrates conceptual underlining of the three classes of Alzheimer’s disease. All three are overlapped, in fact mechanistically identical, from the instance of commencement of AD. In all three forms, the disease is driven by *i*Aβ generated independently of AβPP in an autonomously self-sustainable pathway, i.e., the AD Engine. The pathway’s autonomy is due to the maintenance of *i*Aβ at the above-the-T1 levels and the consequent propagation of the ISR. Conventional AD is triggered by AβPP-derived *i*Aβ at the above-T1 levels; consequently, in this form of AD, the AβPP-independent *i*Aβ generation pathway is autonomously self-sustainable from the moment of its activation. Unconventional activation of the AβPP-independent *i*Aβ generation pathway (by stimulants distinct from AβPP-derived *i*Aβ) in both unconventional forms of AD always occurs at below-T1 *i*Aβ levels, and the pathway attains self-sustainability only after a lag-period when levels of *i*Aβ (produced predominantly in the AβPP-independent pathway) cross the T1 threshold.

The notions of conventional and unconventional AD and the interpretations of both forms of the condition can be combined in the consolidated theory of conventional and unconventional Alzheimer’s disease referred here to as the Expanded Amyloid Cascade Hypothesis 2.0, ACH2.0/E. The ACH2.0/E incorporates ACH2.0, with the latter rendered a special case of the former. The “C” in the “ACH2.0/E ” has two connotations. First, it denotes a defined sequence of events (a “cascade”) initiated by *i*Aβ, such as, for example, formation of NFTs. The second meaning is that *i*Aβ initiates and maintains a cascade of its own generation in the AβPP-independent manner, with newly produced *i*Aβ coercing more of the same (i.e., “the AD Engine” defined above). The etiology of conventional AD and of both classes of unconventional AD, as well as the mechanism sustaining the disease in both conventional and unconventional forms (the AD Engine), is illustrated diagrammatically in Figure 5.

### 9.3. In Unconventional AD the AβPP-Independent iAβ Generation Pathway Attains Self-Sustainability Only after a Lag-Period

Beside the manner of activation of the AβPP-independent *i*Aβ production pathway, there is another principal distinction between conventional and unconventional Alzheimer’s disease. In conventional AD, the AβPP-independent *i*Aβ generation pathway is self-sustainable the moment it becomes operational. This is because, at the time of its activation, the levels of *i*Aβ produced by AβPP proteolysis have already crossed the T1 threshold (the event, which triggered activation of the AβPP-independent *i*Aβ production pathway in the first place). *i*Aβ produced independently of AβPP maintains its pool over the T1 threshold, and thus assures the continuous operation of the AβPP-independent pathway of its own generation. The production of the initial stressor, i.e., AβPP-derived *i*Aβ, becomes redundant at this point. If it were stopped, e.g., by ACH-based drugs, this would be inconsequential; operation of the AβPP-independent *i*Aβ production pathway would continue uninterrupted. 

In unconventional AD this aspect is very different. At the time of unconventional initiation of the AβPP-independent *i*Aβ production pathway (by stimuli distinct from AβPP-derived *i*Aβ) levels of AβPP-derived *i*Aβ are always below the T1 threshold. Indeed, if this were not the case, the AβPP-independent *i*Aβ generation pathway would be activated and the disease would commence in the conventional manner with the crossing of the T1 threshold. Therefore, when the AβPP-independent *i*Aβ generation pathway is activated in either the ISR-dependent or -independent unconventional mode, it is not self-sustainable. If, at this point, the initial unconventional ISR-eliciting stressor or an “activator” of the AβPP-independent *i*Aβ production pathway were withdrawn, operation of the pathway would cease. The condition for the attainment of the self-sustainability is a sufficient duration of the presence of the initial stressor or activator (i.e., “unconventional stimuli”), which would allow *i*Aβ produced in the AβPP-independent pathway to accumulate over the T1 threshold; only then would the pathway become autonomous, and the disease would commence (further discussed and illustrated below).

### 9.4. Unconventional AD Initiated by an ISR-Unrelated Activator of AβPP-Independent iAβ Production May Be Unfeasible

It should be commented that feasibility of the occurrence of the second class of unconventional AD (initiated by an ISR-unrelated activator) depends on the nature of a mechanism underlying the operation of the AβPP-independent *i*Aβ production pathway; such mode of activation appears, in fact, implausible. Indeed, two of the potential mechanisms capable of producing *i*Aβ independently of AβPP, namely the site-specific cleavage of the intact AβPP mRNA and asymmetric RNA-dependent amplification of AβPP mRNA (discussed above and elsewhere [7]), involve uncapped C100-encoding mRNAs. These mRNA species would, presumably, be translatable under the ISR conditions, but not necessarily under the regular physiological circumstances. If and when the AβPP-independent *i*Aβ generation pathway is initiated directly by an ISR-unrelated activator, the ISR is not in effect. If C100 mRNA were capped, the resulting *i*Aβ would trigger the ISR, but then C100 mRNA would likely be rendered untranslatable (as a consequence of the ISR), and operation of the AD Engine would possibly cease. 

If C100-encoding mRNA were uncapped, and were thus untranslatable in the absence of the ISR, no *i*Aβ would be produced independently of AβPP. Consequently, the ISR would not be elicited, the AβPP-independent *i*Aβ generation pathway would not become self-sustainable, and the disease would not occur. In other words, for the ISR-unrelated “activator” pathway to be operative in AD, C100-encoding mRNA should be translatable both in the absence of the ISR and under the ISR conditions, which is rather implausible. This mRNA species is more likely to be uncapped [4,7], and both its generation and its translation might require the ISR conditions. It is possible, therefore, that Alzheimer’s disease, both conventional and unconventional, can be initiated, in distinct manners, only via elicitation of the neuronal ISR.

### 9.5. Additional Definitions: “Conventional” or “Unconventional” Activation of the AβPP-Independent iAβ Production Pathway Defines Conventionality or Unconventionality of Ensuing AD

It follows from the above considerations that conventionality or unconventionality of AD is defined solely by a manner of activation of the active core of the disease, the AβPP-independent *i*Aβ production pathway. To simplify further discussion, additional definitions need to be introduced at this point. The activation of the AβPP-independent *i*Aβ generation pathway triggered by AβPP-derived *i*Aβ accumulated over the T1 threshold is referred to hereafter as “conventional” activation of the pathway. In contrast, activation of the AβPP-independent *i*Aβ production pathway by a stressor distinct from AβPP-derived *i*Aβ or by an activator unrelated to the ISR is designated henceforth as “unconventional”. Accordingly, stimuli (occurrences or conditions that produce stimulants) and stimulants (stressors eliciting the ISR or activators unrelated to the ISR) effecting activation of the AβPP-independent *i*Aβ production pathway and distinct from AβPP-derived *i*Aβ are denoted as “unconventional stimuli” and “unconventional stimulants”. These designations allow for the accurate definition of a category of the disease. Conventional Alzheimer’s disease is AD initiated by conventional activation of the AβPP-independent *i*Aβ generation pathway, whereas unconventional AD is the disease triggered either by the permanent unconventional activation of the AβPP-independent *i*Aβ production pathway or with the assistance of its transient activation. The latter requires an explanation. As discussed above, an event or condition triggering unconventional activation of *i*Aβ production independently of AβPP can be transient. When a stimulus is no longer present, operation of the AβPP-independent *i*Aβ generation pathway would cease, but not before it elevates the *i*Aβ baseline (but still below the T1 threshold). The resumed accumulation of AβPP-derived *i*Aβ from the new, elevated baseline would accelerate the T1 crossing and hasten the occurrence of AD (this is, apparently, what happens in some practitioners of contact sports: TBI suffered at their 20s results in AD in their 40s, whereas without a TBI event, it would occur much later or not occur at all). Thus, the described scenario is a hybrid one: the disease is triggered by ostensibly conventional crossing of the T1 threshold, which was enabled by unconventional activation and transient operation of the AβPP-independent *i*Aβ production pathway. In accordance with the above definition, such form of the disease is referred to hereafter as unconventional AD; it is further discussed and illustrated in Section 11 below.

## 10. Dynamics of *i*Aβ in Alzheimer’s Disease Caused by Enduring Unconventional Activation of the AβPP-Independent *i*Aβ Generation Pathway

**Figure 6:**
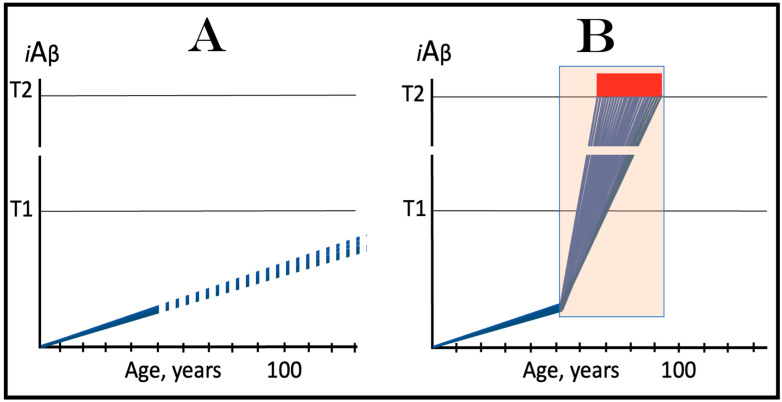
**Dynamics of *i*Aβ accumulation in unconventional AD.** *i*Aβ: Intraneuronal Aβ. ***T1*** *threshold*: Cellular concentration of *i*Aβ that triggers, via eIF2α kinases, and the integrated stress response, activation of the AβPP-independent *i*Aβ generation pathway. ***T2*** *threshold*: Cellular concentration of *i*Aβ, produced predominantly in the AβPP-independent pathway, which triggers apoptosis or necroptosis of neuronal cells. *Blue lines*: Levels of *i*Aβ in individual neurons. *Red Box*: Apoptotic Zone, the continuum of *i*Aβ levels that lead irreversibly to cell death. *Panel* (**A**): Dynamics of *i*Aβ accumulation in a typical individual who would not develop AD unless the AβPP-independent *i*Aβ generation pathway is activated unconventionally. Solid blue lines depict the dynamics of AβPP-derived *i*Aβ accumulation prior to the appearance of unconventional stimulants; dashed blue lines denote the anticipated dynamics of *i*Aβ accumulation in the absence of unconventional stimulants. *Panel* (**B**): Effect of an event or condition that cause long-term presence of unconventional stimulants (the duration of the presence of unconventional stimulants is denoted by the pink box) and, consequently, unconventional activation of the AβPP-independent *i*Aβ generation pathway in neuronal cells. The rate of accumulation of *i*Aβ, now produced predominantly in the AβPP-independent pathway, substantially increases. When its levels cross the T1 threshold, the pathway becomes self-sustainable and the disease commences; when they reach the T2 threshold, the affected neurons commit apoptosis or necroptosis.

The dynamics of accumulation of *i*Aβ in unconventional AD is principally distinct from that taking place in the conventional disease. It is illustrated diagrammatically in Figure 6. Figure 6A depicts the dynamics of *i*Aβ accrual in a typical individual who would not develop conventional AD. *i*Aβ is produced solely in the AβPP proteolytic pathway, its rate of accumulation is low, and its levels would not cross the T1 threshold within the individual’s lifetime unless unconventional AD is triggered. This dynamics, represented by solid lines, is shown until that time when an event occurs or condition develops that causes unconventional AD (dashed lines denote the anticipated dynamics of *i*Aβ accumulation in the absence of unconventional stimuli). At this point, as shown in Figure 6B, the dynamics of *i*Aβ accumulation change drastically. An event or a condition triggers elicitation of the integrated stress response in neuronal cells by means other than AβPP-derived *i*Aβ, the necessary components of the AβPP-independent *i*Aβ generation pathway become available, and the pathway becomes operational (importantly, prior to the T1 crossing). Alternatively, in the second class of unconventional AD, the AβPP-independent *i*Aβ production pathway is activated directly by an ISR-unrelated “activator” (the duration of the presence of unconventional stimulants is denoted by the pink box in Figure 6). In either case, the rate of accumulation of *i*Aβ, now produced predominantly in the AβPP-independent pathway, substantially increases. When its levels cross the T1 threshold, the AβPP-independent iAβ production becomes self-sustainable and the disease commences; when they reach the T2 threshold, the affected neurons commit apoptosis or necroptosis. To cause this chain of events, a condition, say chronic inflammation or infection, which leads to enduring unconventional activation of the AβPP-independent *i*Aβ generation pathway, should persist at least until the levels of *i*Aβ produced in this pathway cross the T1 threshold. This aspect is discussed further in the following section.

## 11. Effects of Transient Unconventional Activation of the AβPP-Independent *i*Aβ Generation Pathway

### 11.1. Transiently and Unconventionally Activated AβPP-Independent iAβ Generation Pathway Attains Self-Sustainability Only If and When Its Product Crosses the T1 Threshold

As discussed above, commencement and progression of AD require sustainable operation of the AβPP-independent *i*Aβ generation pathway. If the latter were to cease, progression of the disease would also stop. In conventional AD, operation of the AβPP-independent *i*Aβ production pathway is always self-sustainable. Moreover, it is self-sustainable from the instance of the pathway’s activation. At this stage, AβPP-derived *i*Aβ is already over the T1 threshold and the ISR is elicited. The rapidly increasing levels of *i*Aβ further propagate the ISR state, which, in turn, supplies the necessary components of the AβPP-independent *i*Aβ production pathway, and thus secures its continuous operation. In unconventional AD, the situation is different. When the AβPP-independent *i*Aβ generation pathway is activated, either via elicitation of the ISR by a stressor other than AβPP-derived *i*Aβ or directly by an ISR-unrelated “activator”, the levels of *i*Aβ are by definition below the T1 threshold (if they were over-T1, it would be a typical case of conventional AD). At this point, two scenarios can be considered. In one, unconventional stimulants (a stressor or “activator”) are present for a long duration and sustain operation of the AβPP-independent *i*Aβ production pathway, as depicted in Figure 6B above. 

**Figure 7:**
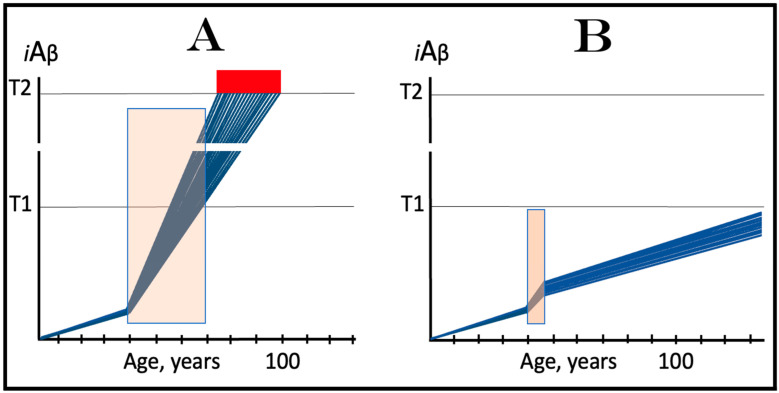
**Effect of transient unconventional activation of the AβPP-independent *i*Aβ generation pathway.** *i*Aβ: Intraneuronal Aβ. ***T1*** *threshold*: Cellular concentration of *i*Aβ that triggers, via eIF2α kinases, and the integrated stress response, activation of the AβPP-independent *i*Aβ generation pathway. ***T2*** *threshold*: Cellular concentration of *i*Aβ, produced predominantly in the AβPP-independent pathway, which triggers apoptosis or necroptosis of neuronal cells. *Blue lines*: Levels of *i*Aβ in individual neurons. *Red Box*: Apoptotic Zone, the continuum of *i*Aβ levels that lead irreversibly to cell death. *Pink Boxes*: Duration of the presence of unconventional stimulants. *Panel* (**A**): The AβPP-independent *i*Aβ generation pathway is activated by unconventional stimulants when the levels of AβPP-derived *i*Aβ are below the T1 threshold (if they were over-T1, it would be a typical case of conventional AD). *i*Aβ generated in this pathway is rapidly accumulating and unconventional stimulants persist for a sufficient duration to allow it to cross the T1 threshold. By virtue of the T1 crossing, the AβPP-independent *i*Aβ production pathway is rendered self-sustainable. This is because, at this point, the pathway is not only supported by an unconventional stimulant, but also sustained by *i*Aβ at over-T1 levels, which propagates the ISR and perpetuates its own production in the AβPP-independent manner. Withdrawal of unconventional stimulant at any time past the T1 crossing would be inconsequential: operation of the AβPP-independent pathway would continue uninterrupted and the disease would progress. *Panel* (**B**): Duration of the presence of unconventional stimulants is too short to allow the T1 crossing. With their withdrawal operation of the AβPP-independent *i*Aβ generation pathway, short of being self-sustainable, would cease. Accumulation of *i*Aβ, now produced solely by the AβPP proteolysis, would continue at the same rate as prior to unconventional activation of the AβPP-independent *i*Aβ generation pathway, but from the elevated baseline. In the depicted scenario, the levels of *i*Aβ do not cross the T1 threshold within the lifetime of an individual, and no AD occurs.

In another scenario, illustrated in Figure 7, the presence of unconventional stimulants within neuronal cells is transient (denoted by pink boxes). What happens after their withdrawal depends on the levels of *i*Aβ, produced predominantly in the AβPP-independent pathway, at the time of the stimulants’ withdrawal. If they are above the T1 threshold, as shown in Figure 7A, the AβPP-independent iAβ production pathway would be self-sustainable and the disease would progress, driven by *i*Aβ produced independently of AβPP, even in the absence of the initial stimulant. In this panel, the AβPP-independent *i*Aβ production pathway has been unconventionally activated. *i*Aβ generated in this pathway has rapidly accumulated, and its levels crossed the T1 threshold in all affected neurons. By virtue of the T1 crossing, the AβPP-independent *i*Aβ production pathway is rendered self-sustainable and AD commences. This is because at this point the pathway is not only supported by an unconventional stimulant, but also sustained by *i*Aβ (at over-T1 levels), which propagates the ISR and perpetuates its own production in the AβPP-independent manner. Withdrawal of unconventional stimulant at any time past the T1 crossing would be inconsequential; operation of the AβPP-independent pathway would continue uninterrupted, and the disease would progress.

If, however, at the time of withdrawal of unconventional stimulants the levels of *i*Aβ are below the T1 threshold, the ISR conditions would not be maintained, and operation of the AβPP-independent *i*Aβ generation pathway, short of being self-sustainable, would cease. Accumulation of *i*Aβ, now produced solely by the AβPP proteolysis, would continue at the same rate as prior to unconventional activation of the AβPP-independent *i*Aβ generation pathway, but from the elevated baseline. In the scenario illustrated in Figure 7B, the levels of *i*Aβ do not cross the T1 threshold within the lifetime of an individual, and no AD occurs. However, as discussed in the following sub-sections, the outcomes of this scenario can be drastically different. 

### 11.2. How a Single TBI Suffered by an Athlete in His 20s Can Lead to AD in His 40s: Unconventional Transient Activation of the AβPP-independent iAβ Production Pathway Accelerates the T1 Crossing by AβPP-derived iAβ and Hastens the Occurrence of AD

**Figure 8:**
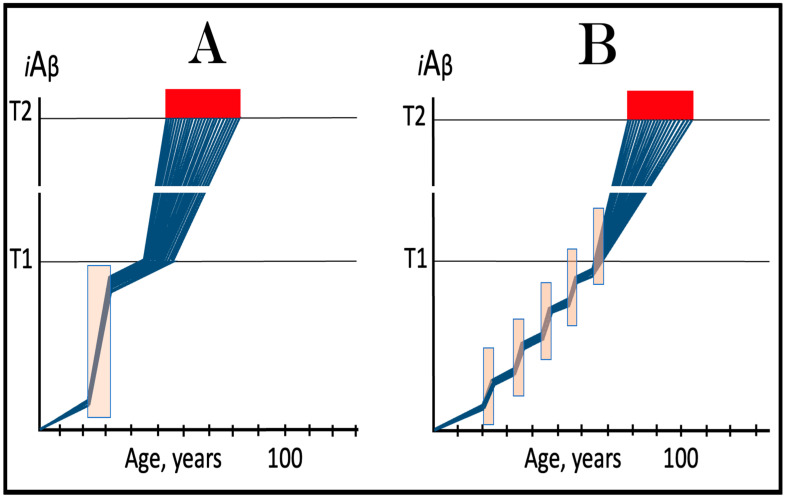
**Transient unconventional activation of the AβPP-independent *i*Aβ generation pathway: Delayed effects.** *i*Aβ: Intraneuronal Aβ. ***T1*** *threshold*: Cellular concentration of *i*Aβ that triggers, via eIF2α kinases, and the integrated stress response, activation of the AβPP-independent *i*Aβ generation pathway. ***T2*** *threshold*: Cellular concentration of *i*Aβ, produced predominantly in the AβPP-independent pathway, which triggers apoptosis or necroptosis of neuronal cells. *Blue lines*: Levels of *i*Aβ in individual neurons. *Red Box*: Apoptotic Zone, the continuum of *i*Aβ levels that lead irreversibly to cell death. *Pink Boxes*: Duration of the presence of unconventional stimulants. *Panel* (**A**): Effect of a single transient unconventional activation of the AβPP-independent *i*Aβ generation pathway. The pathway is activated by an unconventional stimulant, and its operation ceases when it is withdrawn. Following transient operation of the pathway, the baseline of *i*Aβ is elevated, potentially considerably. Accumulation of AβPP-derived *i*Aβ resumes from a high baseline, and would reach the T1 threshold much sooner than in the absence of the transient activity of the AβPP-independent *i*Aβ production pathway. When the T1 threshold is crossed, the self-sustainable AβPP-independent iAβ generation pathway would become operational, and AD would commence. This is, in fact, a “hybrid” yet unconventional situation: the disease is triggered ostensibly “conventionally” (via accumulation of AβPP-derived *i*Aβ), but its occurrence is accelerated, or is enabled, by an “unconventional” transient activation of the AβPP-independent *i*Aβ generation pathway. *Panel* (**B**): Effect of recurrent transient unconventional activations of the AβPP-independent *i*Aβ generation pathway. An individual is subjected to multiple occurrences of an event or condition (or a combination), each resulting in transient activation of the AβPP-independent *i*Aβ generation pathway. In-between these pulses of the pathway’s activity, *i*Aβ continues to accumulate at a “shallow” rate due to its production only in the AβPP proteolytic pathway, but after every pulse, the accumulation resumes from a new baseline, elevated as the result of a “steep” accumulation of *i*Aβ produced in the transiently operative AβPP-independent pathway. If a sufficient number of the pulses of activity of the AβPP-independent pathway occurs, the levels of *i*Aβ would cross the T1 threshold, the autonomous self-sufficient AβPP-independent *i*Aβ would become operational, and the disease would commence.

The notion that a transient occurrence in neuronal cells of a stressor distinct from AβPP-derived *i*Aβ and capable of eliciting the integrated stress response and thus activating generation of *i*Aβ independently of AβPP (or of a direct ISR-unrelated “activator” described above) results in an equally transient operation of the AβPP-independent *i*Aβ production pathway has far-reaching implications. The consequences of such occurrences are illustrated in Figure 8, with durations of the presence of unconventional stimulants and, consequently, of transient operation of the AβPP-independent *i*Aβ generation pathway denoted as pink boxes. Figure 8A depicts the outcome of one-time-only transient unconventional activation of the AβPP-independent *i*Aβ generation pathway. The pathway is activated by the presence of either an unconventional stressor or an “activator”, and its operation ceases when they are withdrawn. However, as the result of transient operation of the pathway, the baseline of *i*Aβ is elevated, potentially considerably. *i*Aβ, now produced only in the AβPP proteolytic pathway, continues to accumulate (from a high baseline) and would reach the T1 threshold much sooner than in the absence of the pulse of activity of the AβPP-independent *i*Aβ production pathway. When the T1 threshold is crossed, the self-sustainable AβPP-independent iAβ generation pathway would become operational, and AD would commence and progress. This is, in fact, a “hybrid” yet unconventional situation: the disease is triggered ostensibly “conventionally” (via accumulation of AβPP-derived *i*Aβ), but its occurrence is accelerated, in fact enabled, by an “unconventional” (i.e., via a stressor distinct from AβPP-derived *i*Aβ or a direct activator of AβPP-independent *i*Aβ production) transient activation of the AβPP-independent *i*Aβ generation pathway.

### 11.3. Why It Can Take Multiple TBIs to Cause AD: Repeated Incidents Result in Step-Wise Elevation of the iAβ Baseline via Multiple Unconventional Transient Activations of the AβPP-Independent iAβ Generation Pathway

The situation considered in Figure 8B is conceptually different. In it, an individual is subjected to multiple occurrences of an event or condition (or a combination of events and conditions) resulting in multiple transient unconventional activations of the AβPP-independent *i*Aβ generation pathway. In-between these pulses of the pathway’s activity *i*Aβ continue to accumulate at a “shallow” rate due to its production only in the AβPP proteolytic pathway. However, after every pulse of rapid accumulation of *i*Aβ transiently produced in the AβPP-independent pathway, the “shallow” *i*Aβ accumulation resumes from a new, significantly elevated baseline. If a sufficient number of the pulses of activity of the AβPP-independent pathway occur, the levels of *i*Aβ would cross the T1 threshold. It is possible that the T1 crossing would occur in a “hybrid” mode discussed above, i.e., during a period of the “shallow” *i*Aβ accumulation; in such a case, AβPP-independent production of *i*Aβ and the disease would be initiated ostensibly “conventionally”, but with the enabling assistance of multiple unconventional transient activations of the AβPP-independent *i*Aβ generation pathway. However, as shown in Figure 8B, with the multiple and regular or semi-regular unconventional activations of the AβPP-independent *i*Aβ generation pathway, it is probable that the T1 crossing would occur during one of the pulses of the pathway’s activity; in such a case, AD would be triggered purely unconventionally, and the T1 crossing would render the pathway self-sustainable.

The scenarios considered in Figure 8A,B are not purely hypothetical, but apparently reflect the real life occurrences. The former provides a plausible description of what happens following a single event such as traumatic brain injury or transient occurrence of a condition capable of unconventional activation of the AβPP-independent *i*Aβ production pathway. This event or condition is not followed immediately by AD. But by significantly elevating the baseline of *i*Aβ, via its transient production in the AβPP-independent pathway, they accelerate the crossing of the T1 threshold and hasten the occurrence of the disease. The latter scenario (shown in panel B) plausibly accounts for unconventional AD caused by multiple events (such as multiple TBIs or chronic traumatic encephalopathy, CTE) or multiple transient occurrences of a condition (or combined events and conditions) that are capable of triggering unconventional activation of AβPP-independent pathway of generation of *i*Aβ; when it crosses the T1 threshold, the pathway becomes self-sustainable and AD commences. 

## 12. Therapeutic Strategies in Conventional AD: ACH-Based Drugs

### 12.1. Preventive Application of the ACH-Based Drugs

**Figure 9:**
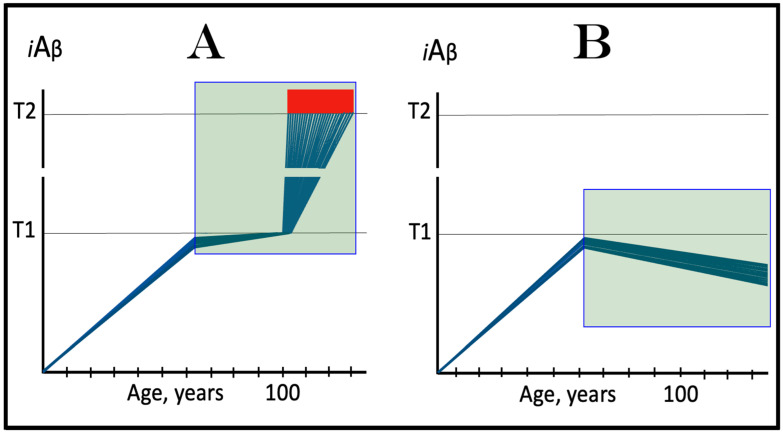
**ACH-based drugs in prevention of conventional AD.** *i*Aβ: Intraneuronal Aβ. ***T1*** *threshold*: Cellular concentration of *i*Aβ that triggers, via eIF2α kinases, and the integrated stress response, activation of the AβPP-independent *i*Aβ generation pathway. ***T2*** *threshold*: Cellular concentration of *i*Aβ, produced predominantly in the AβPP-independent pathway, which triggers apoptosis or necroptosis of neuronal cells. *Blue lines*: Levels of *i*Aβ in individual neurons. *Red Box*: Apoptotic Zone, the continuum of *i*Aβ levels that lead irreversibly to cell death. *Green Boxes*: Duration of treatment with an ACH-based drug. The treatment commences prior to the crossing of the T1 threshold when the AβPP-independent *i*Aβ generation pathway is yet inoperative, and *i*Aβ is derived solely by AβPP proteolysis. The interference with AβPP-derived *i*Aβ accumulation would delay or prevent the crossing of the T1 threshold and, consequently, the commencement and occurrence of AD. *Panel* (**A**): The rate of the accumulation of AβPP-derived *i*Aβ is lowered, but its accumulation continues; eventually, it would, despite the presence of the drug, cross the T1 threshold, thus triggering elicitation of the ISR, activation of the AβPP-independent *i*Aβ generation pathway, and commencement of AD. All this, however, would be delayed by the treatment. *Panel* (**B**): Decrease in the influx of AβPP-derived *i*Aβ is sufficient to reverse the rate of its accumulation. The T1 threshold would not be crossed, the AβPP-independent *i*Aβ generation pathway would not be activated, and the disease would not occur for the duration of the treatment.

As discussed in the preceding sections above, ACH-based AD drugs were designed for and shown to be capable of a substantial reduction of extracellular Aβ. This can occur either by sequestering extracellular Aβ (e.g., with monoclonal antibodies) or by preventing its production and, subsequently, secretion in the AβPP proteolytic/secretory pathway (e.g., with BACE1 inhibitors). By doing this, ACH-based AD drugs accomplish something they were not designed for, namely the reduction, possibly even reversal, of the rate of accumulation of AβPP-derived *i*Aβ. Indeed, smaller pools of extracellular Aβ would translate into its reduced cellular uptake, and utilization of BACE inhibitors would result not only in the diminished extracellular pools of Aβ, but also in the reduced intraneuronal retention of *i*Aβ produced by AβPP processing on the intracellular membranes. The reduced influx of AβPP-derived *i*Aβ would result, in turn, in the reduced rate in its accumulation. If the latter is of a sufficient degree, the outcome could be not only the slower rate of AβPP-derived *i*Aβ accumulation, but, possibly, its reversal if the rate of its physiologically ongoing clearance, i.e., its efflux, exceeds its influx. Since in the ACH2.0 paradigm conventional AD is triggered when AβPP-derived *i*Aβ crosses the T1 threshold, the reduction in the rate of its accumulation would result in the delay of the T1 crossing and, consequently, of the occurrence of the disease. If the rate of accumulation of AβPP-derived *i*Aβ is reversed, neither AβPP-derived *i*Aβ would cross the T1 threshold, nor AD would occur for the duration of the treatment. Such protective and even preventive potential of ACH-based drugs is illustrated in Figure 9A,B (green boxes denote the duration of administration of an ACH-based drug).

### 12.2. ACH-Based Drugs Are Completely Ineffective in Advanced Symptomatic AD

**Figure 10:**
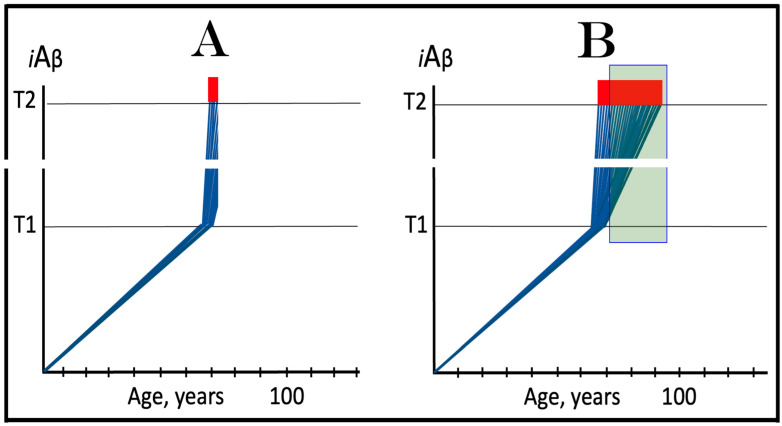
**ACH-based drugs in symptomatic conventional AD.** *i*Aβ: Intraneuronal Aβ. ***T1*** *threshold*: Cellular concentration of *i*Aβ that triggers, via eIF2α kinases, and the integrated stress response, activation of the AβPP-independent *i*Aβ generation pathway. ***T2*** *threshold*: Cellular concentration of *i*Aβ, produced predominantly in the AβPP-independent pathway, which triggers apoptosis or necroptosis of neuronal cells. *Blue lines*: Levels of *i*Aβ in individual neurons. *Red Box*: Apoptotic Zone, the continuum of *i*Aβ levels that lead irreversibly to cell death. *Green Boxes*: Duration of treatment with an ACH-based drug. *Panel* (**A**): Levels of *i*Aβ in the individual neurons of AD patient; the initial state at the start of drug’s administration. AβPP-derived *i*Aβ has crossed the T1 threshold triggering elicitation of the ISR and activation of the AβPP-independent *i*Aβ generation pathway in all affected neurons. A fraction of the neurons has reached the T2 threshold, triggering apoptosis, and AD symptoms have manifested. *Panel* (**B**): Evolution of the initial state in the ACH-based drug-treated AD patient. The influx of AβPP-derived *i*Aβ is reduced, and the rate of its accumulation is lowered or reversed. Its contribution into the cellular *i*Aβ pool at this point, however, is marginal and inconsequential because, at this stage, *i*Aβ is generated predominantly in the AβPP-independent pathway. *i*Aβ produced independently of AβPP continues to accumulate unimpeded and crosses the T2 threshold. The disease progresses and, with a sufficient fraction of neurons lost, enters the end stage.

The situation described in the preceding sub-section changes drastically in advanced symptomatic AD. ACH-based drugs have no therapeutic potential whatsoever at this stage of the disease. Indeed, by the time advanced symptoms of AD manifest, all affected neurons of a patient have crossed the T1 threshold. As the result, the PKR and/or HRI kinases have been activated, eIF2α phosphorylated at Ser51, the integrated stress response elicited, and the AβPP-independent *i*Aβ generation pathway initiated. Operation of the latter is self-sustainable since, at this point, the *i*Aβ levels are above the T1 threshold, and it renders the output of *i*Aβ production by AβPP proteolysis marginal and irrelevant for the progression of the disease. Neither the reduction in the rate of the accumulation of AβPP-derived *i*Aβ, nor even the complete cessation of its production would have any effect on operation of the AβPP-independent iAβ generation pathway. ACH-based drugs, if employed at these stages of AD, would be no less effective in slowing down or reversing accumulation of AβPP-derived *i*Aβ than when utilized preventively. However, at this point, they can neither affect production of *i*Aβ in the AβPP-independent pathway, nor interfere with the progression of AD. This scenario is illustrated in Figure 10. Figure 10A shows the initial state at the time of administration of an ACH-based drug. The levels of AβPP-derived *i*Aβ have crossed the T1 threshold, the self-sustainable AβPP-independent *i*Aβ production pathway was activated, and AD symptoms have manifested. Figure 10B depicts the evolution of the initial state in the presence of an ACH-based drug (green box). The drug has no impact whatsoever, except potentially detrimental side effects. This outcome was indeed observed in multiple clinical trials of ACH-based drugs in advanced symptomatic AD.

### 12.3. Effect of ACH-Based Drugs in Early Symptomatic AD Can Be Only Marginal: Cases of Lecanemab and Donanemab 

**Figure 11:**
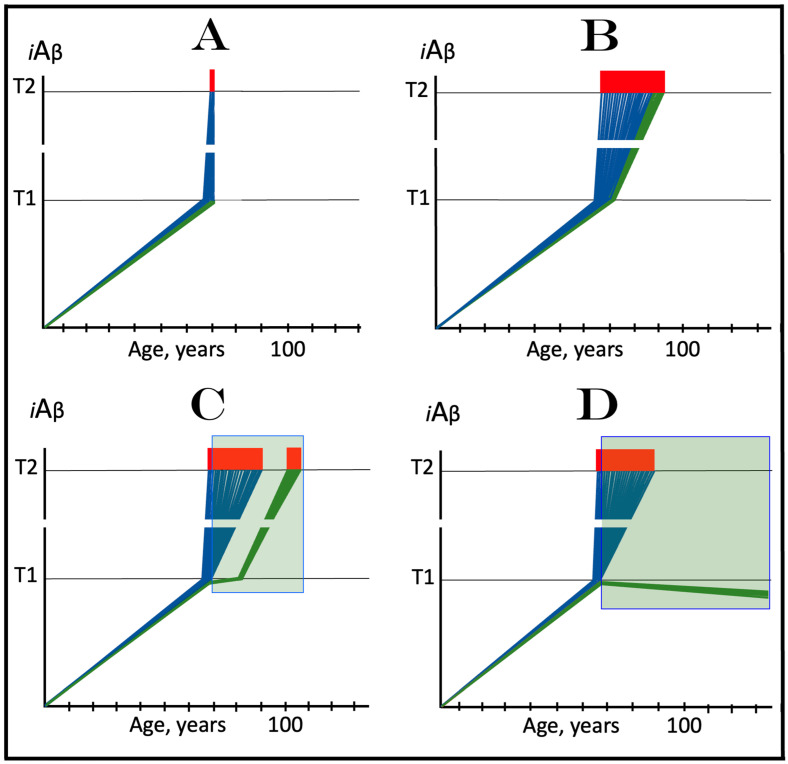
**Effect of the ACH-based drugs in early symptomatic conventional AD can be only marginal: Cases of lecanemab and donanemab**. *i*Aβ: Intraneuronal Aβ. ***T1*** *threshold*: Cellular concentration of *i*Aβ that triggers, via eIF2α kinases, and the integrated stress response, activation of the AβPP-independent *i*Aβ generation pathway. ***T2*** *threshold*: Cellular concentration of *i*Aβ, produced predominantly in the AβPP-independent pathway, which triggers apoptosis or necroptosis of neuronal cells. *Blue lines*: Levels of *i*Aβ in individual neurons. *Green lines*: Neurons with AβPP-derived *i*Aβ below the T1 threshold at the start of the drug’s administration. *Red Box*: Apoptotic Zone, the continuum of *i*Aβ levels that lead irreversibly to cell death. *Green Boxes*: Duration of treatment with an ACH-based drug. *Panel* (**A**): Levels of *i*Aβ in individual neurons of a person; the initial state at the start of the treatment. AβPP-derived *i*Aβ has crossed the T1 threshold and triggered activation of the AβPP-independent *i*Aβ generation pathway in the bulk of neurons but a small fraction of neurons remains sub-T1. *Panel* (**B**): Evolution of the initial state in the untreated individual. The initial sub-T1 fraction crosses the T1 unimpeded; the AβPP-independent *i*Aβ generation pathway becomes operational in all affected neurons, and AD progresses toward the end stage. *Panels* (**C**,**D**): Evolution of the initial state in the treated individual. In both panels, neurons with the operational AβPP-independent *i*Aβ production pathway are insensitive to the treatment; the drug affects only the sub-T1 fraction. *Panel* (**C**): In the initial sub-T1 neuronal fraction, the drug lowers the influx of AβPP-derived *i*Aβ; its accumulation continues at a decreased rate, and it eventually crosses the T1 threshold. The AβPP-independent *i*Aβ generation pathway is activated, and the neurons become unresponsive to the drug; they would reach the Apoptotic Zone but after a certain delay. *Panel* (**D**): In the initial sub-T1 neuronal fraction, the drug-mediated reduction in the influx of AβPP-derived *i*Aβ is sufficient to reverse its accumulation; its cellular levels would be steadily declining, and the T1 threshold would not be reached for the duration of the treatment. Thus, this neuronal fraction would be redeemed, but because it is marginal, so would the overall effect be.

Recently, statistically valid yet marginal therapeutic effects were observed in clinical trials of two ACH-based drugs, lecanemab and donanemab [213,214,215,216,217]. Since these trials were carried out with symptomatic AD patients, it could be argued that the observed outcomes contradict conclusions of the preceding sub-section. This, however, is not the case; in fact, both cases constitute proverbial exceptions that prove the rule. The interpretation of the observed effects of lecanemab and donanemab in the ACH2.0 perspective, first offered in [3], is illustrated in Figure 11. With both drugs, the results obtained were purely preventive (on the cellular level) and due entirely to the timing of the drug’s administration. In the preceding clinical trials of ACH-based drugs, subjects exhibited relatively advanced symptoms of AD, with the AβPP-independent *i*Aβ generation pathway operational in all affected neurons. In contrast, in clinical trials of lecanemab and donanemab, new, very early, markers of the disease were utilized. This resulted in the selection of participants at the very early stages of AD. Because of this, at the time of drugs’ administration, the levels of AβPP-derived *i*Aβ have not yet crossed the T1 threshold in a minor fraction of the affected neurons of individual patients; these neurons (shown as green lines in Figure 11) were responsive to the drugs in the same “preventive” manner as described in Section 12.1 and illustrated in Figure 9 above (the duration of drugs’ administration is denoted as green boxes in Figure 11). The bulk of the affected neurons in individual patients have already crossed the T1 threshold, and thus have “committed” to uninterrupted operation of the AβPP-independent *i*Aβ generation pathway and progression of the cellular AD pathology; these neurons were completely unresponsive to the drugs. The bottom line is that the drugs were effective only preventively and only in the “uncommitted” sub-T1 neurons, where they either delayed or prevented the crossing of the T1 threshold and commencement of the cellular AD pathology (Figure 11C,D), but not curatively, in the over-T1 “AD-committed” neuronal cells (denoted by blue lines). Accordingly, the effect of the drugs in these trials was marginal because, in individual patients, the sub-T1 neuronal populations responsive to the drugs were equally marginal. It needs to be commented that in this interpretation, the effects exhibited by both drugs are not due to their “special” nature. To the contrary, it can be stated with a degree of certainty that any typical ACH-based drug would be similarly effective if administered at similarly early stage of the disease. It can also be stated with equal certainty that, even if administered at the early symptomatic stage of AD, ACH-based drugs could have effect no greater than marginal.

## 13. Therapeutic Strategies in Conventional AD: ACH2.0-Based Drugs

### 13.1. ACH2.0-Based Drugs for Alzheimer’s Disease: A Definition

In the ACH2.0 paradigm, a potential therapeutic strategy for conventional AD follows from the interpretation of the disease. The disease is driven by *i*Aβ generated in the AβPP-independent pathway, which is activated when AβPP-derived *i*Aβ crosses the T1 threshold. Following its activation, this pathway is self-sustainable and is propagated and perpetuated by *i*Aβ at the levels exceeding the T1 threshold. It follows that if the levels of *i*Aβ were depleted to those below the T1 threshold, operation of the AβPP-independent *i*Aβ generation pathway would cease and progression of the disease would stop. Accordingly, the ACH2.0-based drugs can be defined as the agents capable of the targeted degradation and, consequently, depletion of *i*Aβ. The uniqueness and the greatest value of these drugs is that they can be effectively implemented at any stage of symptomatic AD. They can also be used preventively. Conceptually, in their preventive implementation, they achieve the same objective as the ACH-based drugs, namely, a delay or prevention in the crossing of the T1 threshold by AβPP-derived *i*Aβ. However, the ACH2.0-based drugs would be much more effective in attaining this objective because they would deplete *i*Aβ by active degradation, whereas ACH-based drugs do it only indirectly, relying on the physiologically ongoing *i*Aβ clearance processes. Because of their inefficiency, ACH-based drugs have to be administered long-term; indeed, in the lecanemab and donanemab clinical trials, their withdrawal abrogated their beneficial effect [217]. On the other hand, as described below, the ACH2.0-based AD drugs can be administered transiently, both preventively and curatively, with beneficial effects lasting, potentially, for the remaining lifetime of an individual.

### 13.2. Transient Depletion of iAβ by Its Targeted Degradation: Activators of Intra-iAβ-Cleaving Capabilities of BACE1 and/or BACE2

As discussed above, any agent capable of a substantial reduction in the levels of *i*Aβ would constitute a potential ACH2.0-based AD drug. Two prime candidates for this role, both capable of targeted degradation of *i*Aβ, are BACE1 and BACE2. They both possess activities, distinct in BACE1 and BACE2, capable of cleaving within *i*Aβ; in BACE1, such activity is ancillary, whereas in BACE2, it is the major one. Both intra-*i*Aβ-cleaving activities are physiologically relevant: one, when physiologically enhanced (by the Icelandic Aβ mutation), protects from AD, whereas another, when physiologically weakened (by the Flemish Aβ mutation), causes the disease.

#### 13.2.1. Physiologically Occurring Enhancement of Intramolecular Cleavage of *i*Aβ Protects from AD: The Icelandic Mutation

The Icelandic mutation [25,26] protects from AD (and from aging-associated cognitive decline, AACD) by increasing the efficiency of the cleavage by BACE1 or BACE1-associated activity at the alternative site (β’) ten amino acids residues downstream from the primary BACE1-cleaving site (β). The protective nature of the Icelandic Aβ mutation is not the only indication of beneficial effect of the increased rate of cleavage at the β’ site. Thus, exogenous overexpression of BACE1 in animal models significantly increased the rate of cleavage at the β’ site; it also significantly elevated the proportion of β’-cleaved versus full-size Aβ, and substantially decreased the deposition of Aβ in animals’ brains [218,219,220,221]. Moreover, BACE1 (or associated activity) was also shown to be capable of cleaving human *i*Aβ at another alternative site situated between residues 34 and 35 [222,223,224,225]; this produces an intermediate in the physiologically occurring clearance of *i*Aβ. Cleavage at this site was substantially increased by the overexpression of BACE1 [224].

#### 13.2.2. Physiologically Occurring Suppression of Intramolecular Cleavage of *i*Aβ Causes AD: The Flemish Mutation

As mentioned above, cleavages at the β’ site and at the position 34/35 of human *i*Aβ are the ancillary activities of BACE1. In contrast, cleavages at residues 19 and 20 (both phenylalanines) within human *i*Aβ are the major activity of BACE2 (it is also capable of cleaving at the β-site) [226]. It appears that this activity is a physiological control/protection mechanism limiting production of Aβ and the levels of *i*Aβ [227]. Thus, suppression of BACE2 activity in experimental models resulted in a significant increase in the production of Aβ [227], a phenomenon apparently underlying the early onset of AD in the carriers of Flemish mutation. This mutation, occurring at the residue 21 of Aβ, the position contiguous to the major BACE2 cleavage sites, suppresses the efficiency of the cleavages and results in a substantial increase in the levels of *i*Aβ and the early onset of AD [161]. The notions that the activity of BACE2 is protective and its deficiency is deleterious are strongly supported by the observations made with the human pluripotent stem cells-derived brain organoids. In this model system, the BACE2 activity protected the neurons from Aβ-triggered apoptosis, and its deficiency appeared to be a feature common to both AD pathology and Hirschsprung disease [228].

#### 13.2.3. Activators of Intra-*i*Aβ-Cleaving Capabilities of BACE1 and/or BACE2 Would Constitute Potent AD Drugs; Once-in-a-Lifetime Treatment Could Be Sufficient to Prevent the Disease or Arrest Its Progression

It follows that activation or enhancement of the intra-*i*Aβ-cleaving activities of BACE1 and BACE2 should be capable of the targeted degradation and, consequently, depletion of *i*Aβ. Activation of intra-*i*Aβ-cleaving abilities of either BACE1 or BACE2 alone could be sufficient to attain therapeutically meaningful *i*Aβ depletion, but simultaneous activation of both would potentially result in a substantial synergy. This is because not only do the activities in question target distinct sites within *i*Aβ, but also because they apparently operate in distinct cellular locations [229]. Provided that *i*Aβ depletion is sufficiently effective, the treatment can be transient rather than long-term. As further discussed and illustrated below, any transient depletion prior to the T1 crossing would be beneficial by delaying the crossing, whereas when the AβPP-independent *i*Aβ generation pathway is operational, any depletion below the T1 threshold would cease the operation of the pathway and stop progression of the disease. Remarkably, if the depletion is “deep” enough, a single once-in-a-lifetime treatment could be sufficient both for prevention of AD and for its treatment at symptomatic stages. This is because the disease would not occur or recur until the levels of *i*Aβ would reach or be restored to the T1 threshold. Following transient *i*Aβ depletion treatment, with *i*Aβ produced now only by AβPP proteolysis and resuming its accumulation from a sufficiently low baseline, this can take decades and may not occur within the lifetime of the treated individual.

### 13.3. Transient Depletion of iAβ in Prevention of Conventional Symptomatic AD: Once-in-a-Lifetime Treatment Is Potentially Capable of the Life-Long Protection

**Figure 12:**
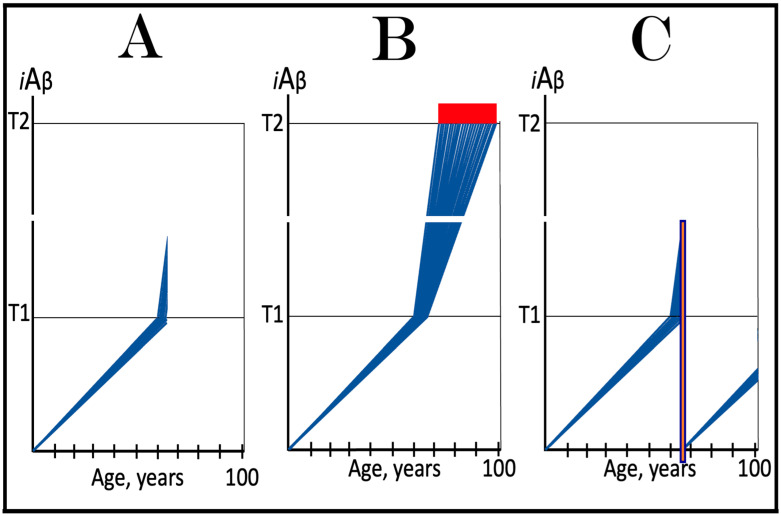
**Prevention of conventional symptomatic AD via transient depletion of *i*Aβ by ACH2.0-based drugs.** *i*Aβ: Intraneuronal Aβ. ***T1*** *threshold*: Cellular concentration of *i*Aβ that triggers, via eIF2α kinases, and the integrated stress response, activation of the AβPP-independent *i*Aβ generation pathway. ***T2*** *threshold*: Cellular concentration of *i*Aβ, produced predominantly in the AβPP-independent pathway, which triggers apoptosis or necroptosis of neuronal cells. *Blue lines*: Levels of *i*Aβ in individual neurons. *Red Box*: Apoptotic Zone, the continuum of *i*Aβ levels that lead irreversibly to cell death. *Orange Box*: Transient depletion of *i*Aβ. *Panel* (**A**): The initial state of the *i*Aβ levels in individual neurons prior to the administration of a drug. In the fraction of the neuronal cells, the levels of *i*Aβ have crossed the T1 threshold and the AβPP-independent *i*Aβ generation pathway has been activated. However, no neurons have yet reached the T2 threshold, and the individual remains asymptomatic. *Panel* (**B**): Evolution of the initial state in the absence of the drug. The remaining sub-T1 neurons cross the T1 threshold and activate the AβPP-independent *i*Aβ production pathway. The levels of *i*Aβ rapidly increase; eventually, they reach and cross the T2 threshold and the disease enters its end-stage. *Panel* (**C**): Evolution of the initial state following the transient *i*Aβ depletion treatment. The treatment substantially depletes *i*Aβ, and its de novo accumulation restarts from a low baseline; no T1 crossing occurs and no AD symptoms develop within the remaining lifespan of the individual.

The scenario depicted in Figure 12 considers the prevention of symptomatic Alzheimer’s disease by ACH2.0 drugs, i.e., by the transient depletion of *i*Aβ through its targeted degradation by activators of intra-*i*Aβ-cleaving capabilities of BACE1 and/or BACE2 or by any other suitable agent. It analyzes the case of an individual who would develop symptomatic AD unless treated preventively. Figure 12A shows the initial state of the *i*Aβ levels in individual neurons prior to administration of a drug. In the fraction of the neuronal cells, the levels of *i*Aβ have crossed the T1 threshold, and in these neurons, the AβPP-independent *i*Aβ generation pathway has been activated. However, no neurons have yet reached the T2 threshold, and the individual remains asymptomatic. This initial state was selected with the purpose of emphasizing the qualitative distinction between the ACH-based and ACH2.0-based drugs. The former are effective only prior to the T1 crossing; with the activation of the AβPP-independent *i*Aβ production pathway, they loose completely their therapeutic potential. Therefore, in this situation, ACH-based drugs could at best protect only a fraction of still sub-T1 neurons. On the other hand, in the same situation, the ACH2.0 drugs can protect the entire neuronal population. Figure 12B shows the evolution of the initial state in the absence of the drug. The remaining sub-T1 neurons cross the T1 threshold and activate the AβPP-independent *i*Aβ production pathway. The levels of *i*Aβ rapidly increase; eventually, they reach and cross the T2 threshold, and the disease enters its end-stage. Figure 12C depicts the evolution of the initial state following the transient *i*Aβ depletion treatment with an ACH2.0-based drug (orange box). The treatment substantially depletes *i*Aβ and its de novo accumulation restarts from a low baseline; no AD symptoms develop. Provided the depletion is sufficiently deep, the levels of *i*Aβ, now produced only by AβPP proteolysis, would not cross the T1 threshold within the lifespan of the individual. Neither the AβPP-independent *i*Aβ generation pathway would be activated, nor would AD occur. Thus, a single, one-time-only transient *i*Aβ depletion treatment by an ACH2.0-based drug has the potential for the life-long protection from AD. 

### 13.4. Transient Depletion of iAβ in Treatment of Conventional Symptomatic AD

As discussed above, in Alzheimer’s disease, *i*Aβ produced in the AβPP-independent pathway fulfills two functions. Firstly, it drives progression of the disease (by achieving levels unattainable to AβPP-derived *i*Aβ). Secondly, by propagating the ISR, it perpetuates the operation of the AβPP-independent pathway of its own production, thus powering the AD Engine (defined above) and making it completely independent from the operation of the AβPP proteolytic pathway and insensitive to ACH-based drugs. Therefore, the key to the efficient intervention in symptomatic AD is abrogating both the function of *i*Aβ, i.e., reducing its levels thus removing the driver of the disease, and terminating its supply by the AβPP-independent mechanism. The transient administration of ACH2.0-based drugs is capable of simultaneously achieving both objectives. Indeed, by accomplishing the former, it attains the latter. With the reduction in the *i*Aβ levels below the T1 threshold, the operation of the AβPP-independent *i*Aβ generation pathway would cease and, likewise, would the progression of AD. *i*Aβ depletion would not affect the AβPP proteolysis, and accumulation of AβPP-derived *i*Aβ would resume de novo. The disease, however, would not recur until and unless the levels of AβPP-derived iAβ are restored to above those of the T1 threshold. It follows that, provided *i*Aβ depletion is sufficiently deep, a single once-in-a-lifetime only treatment with an ACH2.0 drug could confer the lifelong protection from the recurrence of AD. A prognosis for a so-treated patient would largely depend on the timing of the implementation of the treatment and, consequently, on the size of remaining viable neuronal population and its capability for functional recovery.

**Figure 13:**
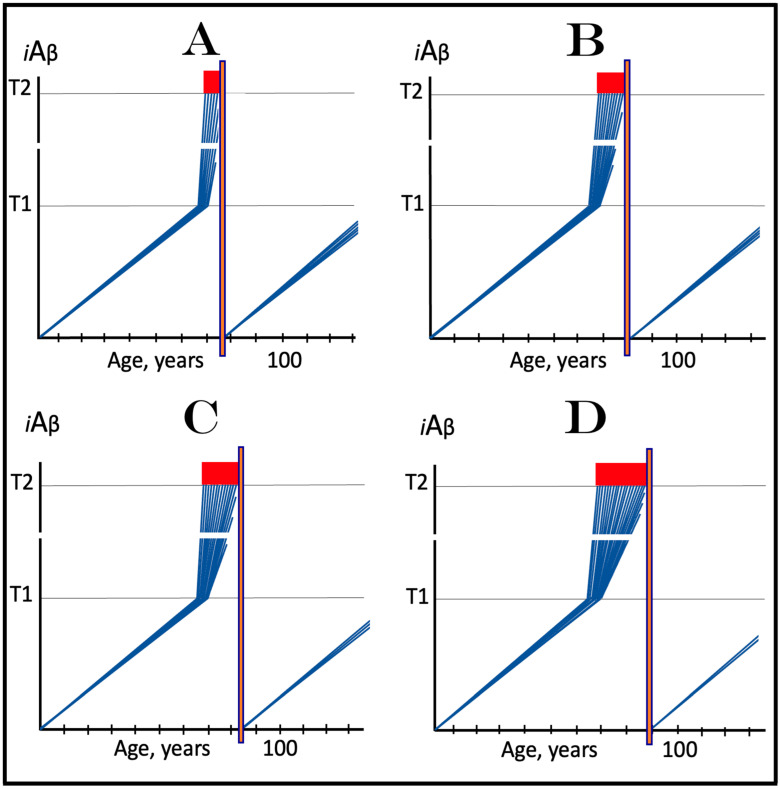
**Treatment of conventional symptomatic AD by transient depletion of iAβ with ACH2.0-based drugs.** *i*Aβ: Intraneuronal Aβ. ***T1*** *threshold*: Cellular concentration of *i*Aβ that triggers, via eIF2α kinases, and the integrated stress response, activation of the AβPP-independent *i*Aβ generation pathway. ***T2*** *threshold*: Cellular concentration of *i*Aβ, produced predominantly in the AβPP-independent pathway, which triggers apoptosis or necroptosis of neuronal cells. *Blue lines*: Levels of *i*Aβ in individual neurons. *Red Box*: Apoptotic Zone, the continuum of *i*Aβ levels that lead irreversibly to cell death. *Orange Boxes*: Transient depletion of *i*Aβ. In panels (**A**–**D**), all affected neurons have crossed the T1 threshold; a fraction of them have also crossed the T2 threshold and AD symptoms have manifested. *Panel* (**A**): The transient depletion treatment is implemented at an early stage of AD. A portion of the affected neurons have crossed the T2 threshold and committed apoptosis; it cannot be redeemed. The bulk of the neurons, however, did not reach the T2 threshold at this early stage; these neurons are redeemable. Following transient treatment by an ACH-2.0-based drug, levels of *i*Aβ are substantially depleted and re-set. The AβPP-independent *i*Aβ generation pathway is rendered inoperative, and progression of the disease ceases. The de novo accumulation of *i*Aβ, powered at this stage only by AβPP proteolysis, resumes from a low baseline. Its levels would not reach the T1 threshold and, consequently, the disease would not recur within the lifespan of the treated individual. *Panels* (**B**–**D**): Effects of transient implementation of the *i*Aβ depletion treatment at progressively advanced stages of the disease. Qualitatively, it is the same as shown in panel (**A**). What differ are the progressively diminished sizes of still-viable neuronal subpopulations at the time of the treatment’s administration. These subpopulations would be redeemed, and the progression of the disease would be stopped or significantly slowed down. However, due to the diminishing redemption of still-viable neurons, the probability of cognitive recovery would be reversely proportional to the advancement of the disease at the time of administration of the treatment.

The above considerations are illustrated diagrammatically in Figure 13. Different panels depict the consequences of implementation of the transient *i*Aβ depletion treatment at different stages of the disease. In Figure 13A, the transient treatment is implemented at an early stage of AD (orange box). A portion of the affected neurons has crossed the T2 threshold and committed apoptosis (or necroptosis); this portion cannot be redeemed. The bulk of the neurons, however, did not reach the T2 threshold at this early stage; these neurons are redeemable. Following transient *i*Aβ depletion treatment by an ACH-2.0-based drug, levels of *i*Aβ are substantially depleted and re-set. The AβPP-independent *i*Aβ generation pathway is rendered inoperative, and progression of the disease ceases. The de novo accumulation of *i*Aβ, powered at this stage only by AβPP proteolysis, resumes from a low baseline. Its levels would not reach the T1 threshold and, consequently, the disease would not recur within the lifespan of the treated individual. 

Figure 13B–D depicts the effects of transient implementation of the *i*Aβ depletion treatment (orange boxes) at progressively advanced stages of the disease. Conceptually, they are the same as shown in Figure 13A. What differs is progressively diminishing sizes of still viable neuronal subpopulations at the time of the treatment’s administration. These subpopulations would be redeemed, and the progression of the disease would be stopped or significantly slowed down. However, due to the diminishing redemption of still viable neurons, the probability of cognitive recovery would be reversely proportional to the advancement of the disease at the time of administration of the *i*Aβ depletion treatment.

## 14. ACH-Based Drugs Cannot Be Effective in Unconventional AD and Are of Limited Utility in Its Prevention

In the above discussion, it has been established that the ACH-based drugs can have no effect on the conventionally activated AβPP-independent *i*Aβ generation pathway. The reason for this is that the latter is self-sustainable and, due to its large output of *i*Aβ, renders the AβPP proteolytic pathway marginal because its contribution to the cellular *i*Aβ pool becomes marginal. The ACH-based drugs are capable of affecting only AβPP-derived *i*Aβ, but even if its influx is completely stopped (by ACH-based drugs), this would have no effect whatsoever on the operation of the AβPP-independent *i*Aβ generation pathway and on the progression of AD. Importantly, the ACH-based drugs can have no effect on the unconventionally activated AβPP-independent *i*Aβ production pathway, either. To simplify the analysis of the effect of ACH-based drugs on the unconventionally initiated AβPP-independent *i*Aβ generation pathway, the duration of operation of the latter can be divided into two temporal periods. In the first period, the pathway is not self-sustainable, and its activity depends on the presence of unconventional stimuli; if these stimuli were withdrawn, operation of the pathway would cease. These stimuli are, by definition, distinct from AβPP-derived *i*Aβ; therefore, suppression of the influx of the latter can have no effect on operation of the unconventionally activated AβPP-independent *i*Aβ production pathway. The second temporal period commences when the levels of *i*Aβ, produced independently of AβPP, cross the T1 threshold. At this point, the pathway becomes self-sustainable, driven by its own product (*i*Aβ), indistinguishable from the conventionally activated AβPP-independent *i*Aβ production pathway, and insensitive to the ACH-based drugs for the reasons discussed above.

**Figure 14:**
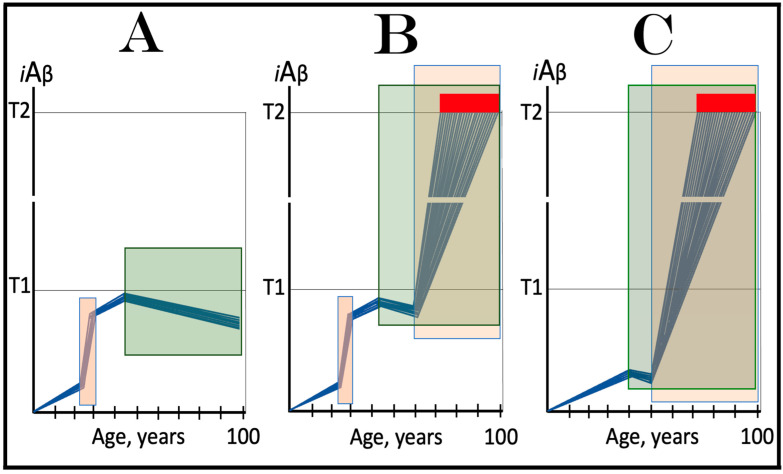
**ACH-based drugs are of limited utility in unconventional AD.** *i*Aβ: Intraneuronal Aβ. ***T1*** *threshold*: Cellular concentration of *i*Aβ that triggers, via eIF2α kinases, and the integrated stress response, activation of the AβPP-independent *i*Aβ generation pathway. ***T2*** *threshold*: Cellular concentration of *i*Aβ, produced predominantly in the AβPP-independent pathway, which triggers apoptosis or necroptosis of neuronal cells. *Blue lines*: Levels of *i*Aβ in individual neurons. *Red Box*: Apoptotic Zone, the continuum of *i*Aβ levels that lead irreversibly to cell death. *Pink Boxes*: Duration of the presence of unconventional stimulants. *Green Boxes*: Duration of administration of an ACH-based drug. *Panel* (**A**): The unconventionally activated AβPP-independent *i*Aβ generation pathway operates transiently, and when its operation ceases, *i*Aβ levels remain sub-T1, but are significantly elevated. Accumulation of AβPP-derived *i*Aβ resumes from the new high baseline, and, in the absence of the treatment, it would eventually cross the T1 threshold, activate self-sustainable AβPP-independent *i*Aβ generation pathway, and initiate AD. If administration of an ACH-based drug commences prior to the T1 crossing, it potentially reverses accumulation of AβPP-derived *i*Aβ, and neither T1 crossing nor AD would occur for the duration of the treatment. *Panel* (**B**): Expansion of the scenario shown in panel (**A**). During treatment with an ACH-based drug an event occurs or a condition arises that cause another unconventional activation of the AβPP-independent *i*Aβ generation pathway. This renders the ACH-based drug ineffective. *i*Aβ produced independently of AβPP crosses the T1 threshold, the AβPP-independent *i*Aβ generation pathway becomes self-sustainable, and AD commences. *Panel* (**C**): Administration of the ACH-based drug commences prior to unconventional activation of the AβPP-independent *i*Aβ production pathway. However, the drug can prevent neither operation of the pathway nor the T1 crossing and commencement of AD. For details, see the main text.

It follows that the only situation where the ACH-based drugs can have preventive effect in unconventional AD is the “hybrid” one, considered in Section 11 above. In this scenario, the unconventionally activated AβPP-independent *i*Aβ generation pathway operates only transiently, and when its operation ceases, *i*Aβ levels remain sub-T1, but are significantly elevated. Accumulation of AβPP-derived *i*Aβ resumes from a new high baseline, and it would eventually cross the T1 threshold, activate the self-sustainable AβPP-independent *i*Aβ generation pathway, and initiate AD. If administration of an ACH-based drug commences prior to the T1 crossing, and if the drug reverses accumulation of AβPP-derived *i*Aβ (the best possible outcome), no T1 crossing and no AD would occur for the duration of the treatment. This scenario is illustrated in Figure 14A (in all panels of Figure 14 pink boxes denote the duration of the presence of unconventional stimulants and green boxes signify the duration of administration of an ACH-based drug). If, however, during the treatment period, an event would occur or a condition would arise that cause another unconventional activation of the AβPP-independent *i*Aβ generation pathway, the ACH-based drug would be rendered ineffective. *i*Aβ produced in the AβPP-independent pathway would cross the T1 threshold, the pathway would become self-sustainable, and AD would commence; this scenario is depicted in Figure 14B. Similarly, as shown in Figure 14C, if administration of the ACH-based drug starts prior to unconventional activation of AβPP-independent *i*Aβ production pathway, the drug would prevent neither operation of the pathway, nor the T1 crossing and commencement of AD.

## 15. ACH2.0-Based Drugs in Unconventional AD

### 15.1. ACH2.0-Based Drugs in Prevention of Unconventional AD

**Figure 15:**
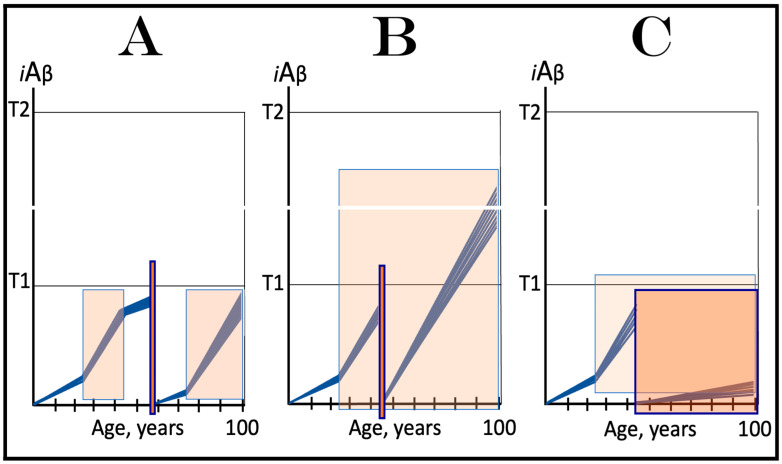
**ACH2.0-based drugs in prevention of unconventional AD.** *i*Aβ: Intraneuronal Aβ. ***T1*** *threshold*: Cellular concentration of *i*Aβ that triggers, via eIF2α kinases and the integrated stress response, activation of the AβPP-independent *i*Aβ generation pathway. ***T2*** *threshold*: Cellular concentration of *i*Aβ, produced predominantly in the AβPP-independent pathway, which triggers apoptosis or necroptosis of neuronal cells. *Blue lines*: Levels of *i*Aβ in individual neurons. *Red Box*: Apoptotic Zone, the continuum of *i*Aβ levels that lead irreversibly to cell death. *Pink Boxes*: Duration of the presence of unconventional stimulants. *Orange Boxes*: Duration of *i*Aβ depletion treatment by ACH2.0 drugs. *Panel* (**A**): The unconventionally activated AβPP-independent *i*Aβ generation pathway operates transiently, and when its operation ceases, *i*Aβ levels remain sub-T1, but are significantly elevated. Accumulation of AβPP-derived *i*Aβ resumes from a new high baseline, and, in the absence of the treatment, it would eventually cross the T1 threshold and trigger AD. Transient treatment with an ACH2.0-based drug substantially depletes *i*Aβ and prevents the T1 crossing potentially for the remaining lifetime of the treated individual. If, however, following the transient *i*Aβ depletion treatment, another unconventional activation and transient operation of the AβPP-independent *i*Aβ generation pathway takes place, it would be in the context of a low *i*Aβ baseline. Consequently, *i*Aβ produced in this pathway may not reach the T1 threshold. In panels (**B**,**C**) unconventional stimulant are present long-term; the AβPP-independent *i*Aβ generation pathway is activated, but *i*Aβ levels do not reach the T1 threshold by the time of implementation of the *i*Aβ-depleting treatment. *Panel* (**B**): The ACH2.0 drug is administered transiently and the levels of *i*Aβ are substantially depleted. Following the withdrawal of the drug, with the unconventional stimulus present, AβPP-independent *i*Aβ generation pathway remains active, and accumulation of its *i*Aβ product would resume from a low baseline. If it crosses the T1 threshold, the pathway would become self-sustainable, and the disease would commence. *Panel* (**C**): The ACH2.0 drug is administered long-term. *i*Aβ is depleted to a low baseline, and its levels are maintained low by the drug. The T1 threshold would not be crossed, and the disease would not occur for the duration of the treatment.

To prevent AD, both conventional and unconventional, the levels of *i*Aβ must be precluded from crossing the T1 threshold. In this, transient administration of the *i*Aβ depletion treatment can achieve, much more efficiently, the same beneficial outcome as ACH-based drugs (administered long-term). Figure 15A considers the effect of the ACH2.0-based drugs in the same situations as shown in Figure 14A,B. In it, following transient activity of the unconventionally initiated AβPP-independent *i*Aβ production pathway (pink box), AβPP-derived *i*Aβ resumes its accumulation from a significantly elevated, yet still sub-T1, baseline, and would cross the T1, thus triggering AD, if the individual is not treated. Transient treatment with an ACH2.0-based drug (orange box) substantially depletes *i*Aβ and prevents the T1 crossing potentially for the remaining lifetime of the treated individual. And even if, following the transient *i*Aβ depletion treatment, an event occurs or a condition arises that trigger unconventional activation and transient operation of the AβPP-independent *i*Aβ generation pathway (second pink box), it would take place in the context of a low *i*Aβ baseline. Consequently, *i*Aβ produced in this pathway may not reach the T1 threshold within the lifetime of an individual (compare this with the situation shown in Figure 14B, where the second unconventional activation of the AβPP-independent *i*Aβ production pathway occurs in the context of a high *i*Aβ baseline).

In addition, the ACH2.0-based drugs can do preventively what ACH-based drugs cannot. This is because whereas the latter affect only AβPP-derived *i*Aβ, the former deplete the totality of *i*Aβ (produced in both AβPP-dependent and –independent pathways. This potential of the ACH2.0 drugs is illustrated in Figure 15B,C. In both panels the unconventional stimulants are present long-term (pink boxes) and the unconventionally activated AβPP-independent *i*Aβ production pathway is operational; in both the levels of *i*Aβ have not yet reached the T1 threshold. In panel B the *i*Aβ depletion treatment (orange box) is implemented transiently. As the result, the levels of *i*Aβ are substantially depleted. Following the withdrawal of the drug, with the unconventional stimulus still present, AβPP-independent *i*Aβ generation pathway remains active, and accumulation of its *i*Aβ product would resume from a low baseline and may not reach the T1. If, eventually, it would cross the T1 threshold, the pathway would become self-sustainable and the disease would commence but the levels of iAβ may not reach the T2 threshold within the lifetime of an individual. In panel C the long-term *i*Aβ depletion treatment (orange box) is implemented. As the result, *i*Aβ is depleted to a low baseline and its levels are maintained low by the drug. The T1 threshold would not be crossed and the disease would not occur for the duration of the treatment. The preventive transient treatment with ACH2.0 drugs, discussed in the present section, can potentially be combined with targeting the initial causes of unconventional AD or the unconventional stimuli activating the AβPP-independent *i*Aβ production pathway. The possible outcomes of such approach are discussed in Section 16 below.

### 15.2. Effect of Transient Depletion of iAβ via Its Targeted Degradation in Unconventional Symptomatic AD

**Figure 16:**
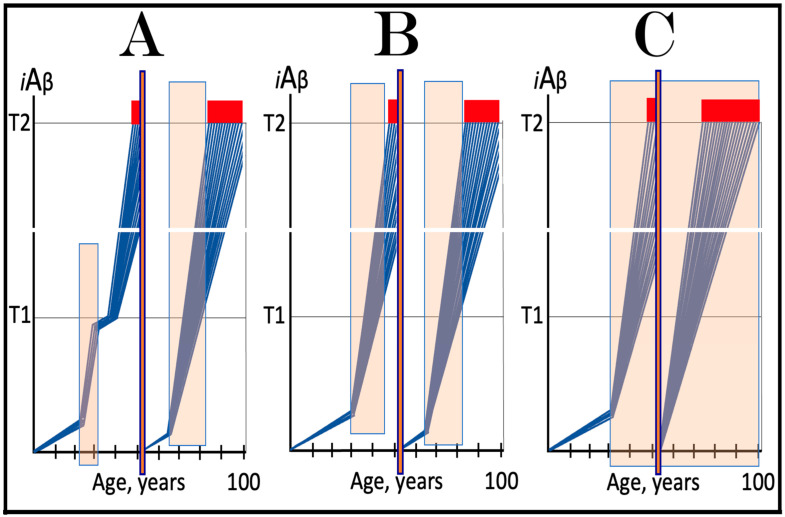
**Transient implementation of ACH2.0-based drugs in treatment of unconventional symptomatic AD.** *i*Aβ: Intraneuronal Aβ. ***T1*** *threshold*: Cellular concentration of *i*Aβ that triggers, via eIF2α kinases and the integrated stress response, activation of the AβPP-independent *i*Aβ generation pathway. ***T2*** *threshold*: Cellular concentration of *i*Aβ, produced predominantly in the AβPP-independent pathway, which triggers apoptosis or necroptosis of neuronal cells. *Blue lines*: Levels of *i*Aβ in individual neurons. *Red Box*: Apoptotic Zone, the continuum of *i*Aβ levels that lead irreversibly to cell death. *Pink Boxes*: Duration of the presence of unconventional stimulants. *Orange Boxes*: Transient depletion of *i*Aβ by ACH2.0 drugs. *Panel* (**A**): Transient activity of the unconventionally activated AβPP-independent *i*Aβ generation pathway significantly elevates the levels of *i*Aβ. When operation of the pathway ceases, accumulation of AβPP-derived *i*Aβ resumes from a high baseline; it crosses the T1 threshold and activates the self-sustainable AβPP-independent *i*Aβ production pathway. AD commences and *i*Aβ levels rapidly increase; when they cross the T2 threshold in a fraction of the neurons, apoptosis ensues and AD symptoms manifest. The transient *i*Aβ depletion treatment administered at this point substantially reduces *i*Aβ levels, and its accumulation resumes solely in the AβPP proteolytic pathway. If no additional unconventional activations of the AβPP-independent *i*Aβ production pathway would take place, *i*Aβ levels would not cross the T1 threshold and the disease would not recur. If, however, following transient administration of an ACH2.0-based drug, another unconventional activation of the AβPP-independent *i*Aβ generation pathway does occur, *i*Aβ would cross the T1 threshold, the AβPP-independent *i*Aβ production pathway would become self-sustainable, and the disease would recur. *Panel* (**B**): Similar to panel (**A**), with identical outcomes. The only difference is that the initial unconventionally activated AβPP-independent *i*Aβ generation pathway remains operational, at least through the crossing of the T1 threshold, and is rendered self-sustainable by the crossing. *Panel* (**C**): Following transient *i*Aβ depletion treatment, the AβPP-independent pathway remains active due to the continuous presence of the unconventional stimulant. *i*Aβ rapidly accumulates, albeit from a low baseline; given sufficient time, it would reach the T1 threshold, and the disease would recur.

In unconventional symptomatic AD, the levels of *i*Aβ have crossed the T1 threshold, and the AβPP-independent *i*Aβ generation pathway has become self-sustainable. Its operation, at this point, is not dependent anymore on the continuous presence of an unconventional stimulus; withdrawal of the latter would have no effect on the former. However, the recurrent transient occurrence or the continuous presence of an unconventional stimulus capable of activating the AβPP-independent *i*Aβ production pathway would significantly affect the effectiveness of the ACH2.0-based drugs at symptomatic stages of the disease. This notion is illustrated in Figure 16. In Figure 16A, transient activity of the unconventionally activated AβPP-independent *i*Aβ generation pathway (pink box) significantly elevated the levels of *i*Aβ. When operation of the pathway ceases, accumulation of AβPP-derived *i*Aβ resumes from a new high baseline. It crosses the T1 threshold and activates the self-sustainable AβPP-independent *i*Aβ production pathway. The disease commences, and *i*Aβ levels rapidly increase; when they cross the T2 threshold in a fraction of the neurons, cells commit apoptosis and AD symptoms manifest. The transient *i*Aβ depletion treatment (orange box) administered at this point would substantially reduce *i*Aβ levels. Because the unconventional stimulus capable of activating the AβPP-independent *i*Aβ production pathway is absent, accumulation of *i*Aβ would resume solely in the AβPP proteolytic pathway. Provided that no additional unconventional activations of the AβPP-independent *i*Aβ production pathway would occur within the remaining lifetime of the patient, *i*Aβ levels would not cross the T1 threshold, and the disease would not recur within the lifespan of the individual, the outcome identical to that shown in Figure 13. However, if, following transient administration of an ACH2.0-based drug, an event would occur or a condition would arise that triggers an unconventional activation and operation of the AβPP-independent *i*Aβ generation pathway (second pink box), *i*Aβ would cross the T1 threshold, the AβPP-independent *i*Aβ production pathway would become self-sustainable, and the disease would recur.

In Figure 16B,C, the unconventionally activated AβPP-independent *i*Aβ generation pathway remains operational, at least through the crossing of the T1 threshold (pink box). With the T1 crossing, the pathway is rendered self-sustainable, the disease commences, and when a fraction of the neurons crosses the T2 threshold, AD symptoms manifest, and the transient *i*Aβ depletion treatment is implemented (orange box). In Figure 16B, by the time of the administration of the ACH2.0-based drug, the unconventional stimulus is no longer present. Consequently, the outcome is identical to that depicted in Figure 16A. Following its transient depletion, accumulation of *i*Aβ, now produced only by AβPP proteolysis, resumes from a low baseline and, provided no additional unconventional activations of the AβPP-independent *i*Aβ production pathway would take place, neither the T1 crossing would occur, nor would AD recur within the lifespan of the treated individual. However, if an additional unconventional activation of the AβPP-independent *i*Aβ generation pathway would occur (second pink box), the T1 threshold would be crossed and AD would recur. In Figure 16C, the unconventional stimulus (pink box) is still present at the time (and past the time) of the transient *i*Aβ depletion treatment (orange box). Following transient depletion treatment, accumulation of *i*Aβ produced in the AβPP-independent pathway, which remains active due to the continuous presence of the unconventional stimulus, would resume, albeit from a low baseline. Given sufficient time, it would reach the T1 threshold, and the disease would recur. This issue can potentially be addressed by simultaneous suppression of the unconventional stimulus, a scenario addressed in Section 16 below.

### 15.3. Effect of Long-Term iAβ Depletion Therapy in Unconventional Symptomatic AD

**Figure 17:**
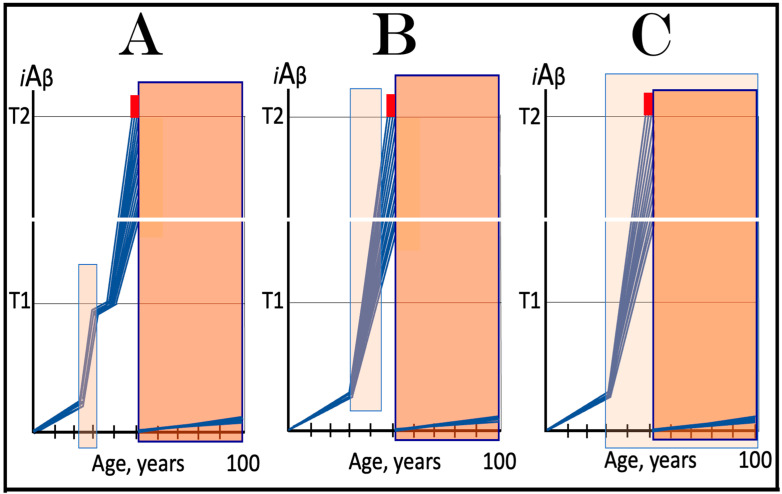
**Long-term implementation of ACH2.0-based drugs in treatment of unconventional symptomatic AD.** *i*Aβ: Intraneuronal Aβ. ***T1*** *threshold*: Cellular concentration of *i*Aβ that triggers, via eIF2α kinases and the integrated stress response, activation of the AβPP-independent *i*Aβ generation pathway. ***T2*** *threshold*: Cellular concentration of *i*Aβ, produced predominantly in the AβPP-independent pathway, which triggers apoptosis or necroptosis of neuronal cells. *Blue lines*: Levels of *i*Aβ in individual neurons. *Red Box*: Apoptotic Zone, the continuum of *i*Aβ levels that lead irreversibly to cell death. *Pink Boxes*: Duration of the presence of unconventional stimulants. *Orange Boxes*: Duration of *i*Aβ depletion treatment by ACH2.0 drugs. *Panel* (**A**): Transient activity of the unconventionally activated AβPP-independent *i*Aβ generation pathway significantly elevates the levels of *i*Aβ. When operation of the pathway ceases, accumulation of AβPP-derived *i*Aβ resumes from a high baseline; it crosses the T1 threshold and activates the self-sustainable AβPP-independent *i*Aβ production pathway. AD commences and *i*Aβ levels rapidly increase; when they cross the T2 threshold in a fraction of the neurons, apoptosis ensues and AD symptoms manifest. The long-term *i*Aβ depletion treatment administered at this point substantially reduces *i*Aβ levels and renders the AβPP-independent *i*Aβ production pathway inoperative. A recurrence of the unconventional activation of the AβPP-independent *i*Aβ production pathway at this stage would be inconsequential because the drug would prevent the resumption of significant *i*Aβ accumulation. *Panel* (**B**): Similar to panel (**A**), with identical outcomes. The only difference is that the initial unconventionally activated AβPP-independent *i*Aβ generation pathway remains operational, at least through the crossing of the T1 threshold, and is rendered self-sustainable by the crossing. *Panel* (**C**): Following commencement of the long-term administration of an ACH2.0-based drug, *i*Aβ is substantially depleted, but the AβPP-independent pathway remains active due to the continuous presence of the unconventional stimulant. Despite this, and due to the presence of the drug, the levels of *i*Aβ would remain low for the duration of the treatment. Note that in all three panels, neither the T1 threshold would be crossed, nor the disease would recur for the duration of the drug’s administration.

The therapeutic strategy discussed in the preceding subsection, i.e., transient *i*Aβ depletion by ACH2.0-based drugs, has obvious limitations. If unconventional activation of the AβPP-independent *i*Aβ generation pathway was only transient and no unconventional stimuli are present at the time of drug’s administration, the treatment could be effective. If, however, another unconventional activation of the AβPP-independent *i*Aβ production pathway occurs downstream from the transient treatment, it would be unimpeded by the treatment. And if the transient *i*Aβ depletion treatment were implemented in the presence of the unconventional stimuli, the AβPP-independent *i*Aβ production pathway would remain active, and the de novo accumulation of *i*Aβ produced in this pathway would start as soon as the drug is withdrawn; the relief would be only temporary. These potential problems are resolved if the *i*Aβ depletion treatment were administered long-term rather than transiently. This strategy is illustrated in Figure 17, which considers the same situations as shown in Figure 16 but under the long-term *i*Aβ depletion treatment. In Figure 17A–C, the *i*Aβ depletion treatment (orange boxes) commences at the symptomatic stages of AD and continues for the long-term duration. As the result, *i*Aβ is substantially depleted. In panels A and B the AβPP-independent *i*Aβ generation pathway is rendered inoperative by the treatment, and the potential recurrence of unconventional activation of the AβPP-independent *i*Aβ production pathway would be inconsequential because the drug would prevent the resumption of significant *i*Aβ accumulation. In panel C, due to the continuous presence of unconventional stimuli (pink box), the AβPP-independent *i*Aβ generation pathway would remain active. However, due to the presence of the drug, the levels of *i*Aβ would remain low for the duration of the treatment. In all three panels, neither the T1 threshold would be crossed, nor would the disease recur as long as the drug is administered.

### 15.4. Effect of Recurrent Transient iAβ Depletion Treatments in Unconventional AD 

As discussed above, any agent capable of the depletion of *i*Aβ via its targeted degradation could constitute an ACH2.0-based drug. The prime candidates for this role are activators of the intra-*i*Aβ-cleaving capabilities of BACE1 and BACE2. The long-term administration of any drug could potentially have detrimental side effects. In case of manipulation of BACE, this danger is apparently more than hypothetical. Indeed, long-term administration of BACE1 inhibitors in clinical trials resulted in severe adverse effects [14,15]. On the other hand, the proposed ACH2.0-based drugs are the activators, not the inhibitors, of the BACE1 and BACE2 capabilities, and it is possible that in these cases no adverse effects would manifest. In fact, this is more than just a possibility. Indeed, the probability that the enhancement of the *i*Aβ-cleaving activity of BACE1 would have little, if any, detrimental effect is strongly suggested by the case of the Icelandic mutation. In the carriers of this mutation, the β’-site cleaving activity of BACE1 (the prime target of the ACH2.0-base drugs) is significantly enhanced, yet the only effect these individuals are experiencing is beneficial: protection from both AD and aging-associated cognitive decline [25,26]. 

**Figure 18:**
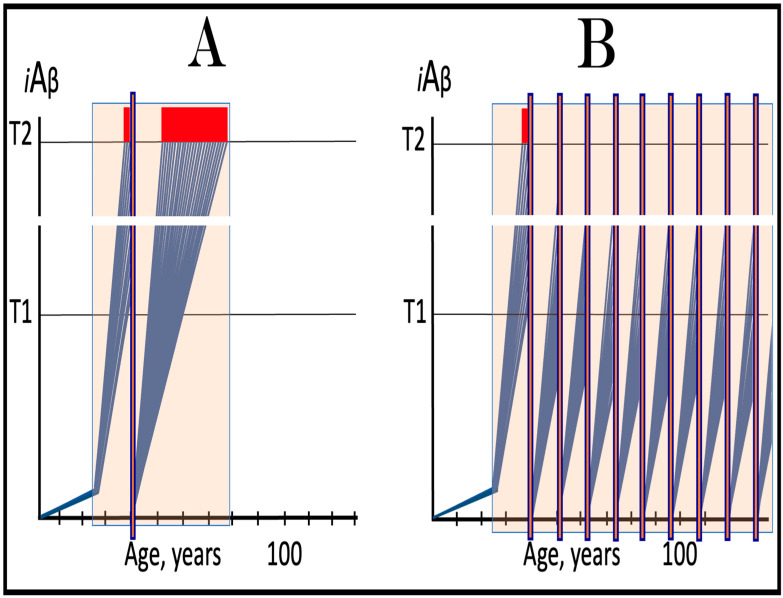
**Recurrent implementation of transient *i*Aβ depletion treatment in unconventional symptomatic AD.** *i*Aβ: Intraneuronal Aβ. ***T1*** *threshold*: Cellular concentration of *i*Aβ that triggers, via eIF2α kinases and the integrated stress response, activation of the AβPP-independent *i*Aβ generation pathway. ***T2*** *threshold*: Cellular concentration of *i*Aβ, produced predominantly in the AβPP-independent pathway, which triggers apoptosis or necroptosis of neuronal cells. *Blue lines*: Levels of *i*Aβ in individual neurons. *Red Box*: Apoptotic Zone, the continuum of *i*Aβ levels that lead irreversibly to cell death. *Pink Boxes*: Duration of the presence of unconventional stimulants. *Orange Boxes*: Transient depletion of *i*Aβ by ACH2.0 drugs. In both panels unconventional stimulants are present tor the long-term duration. *Panel* (**A**): Unconventionally activated AβPP-independent *i*Aβ generation pathway remains operational through and is rendered self-sustainable by the crossing of the T1 threshold. AD commences and *i*Aβ levels rapidly increase; when they cross the T2 threshold in a fraction of the neurons, apoptosis ensues and AD symptoms manifest. The transient *i*Aβ depletion treatment administered at this point substantially reduces *i*Aβ levels. However, following the treatment, the AβPP-independent pathway remains active due to the continuous presence of the unconventional stimulant. *i*Aβ rapidly accumulates; given sufficient time, it reaches the T1 threshold, and the disease recurs. Note that a single transient administration of the drug buys some disease-free time (between the withdrawal of the drug and the subsequent T1 crossing), which is measured in years. *Panel* (**B**): Effect of concurrent transient administrations of the ACH2.0-based drug. The initial transient *i*Aβ depletion treatment is administered, as in panel (**A**), at the early symptomatic stages of AD. Each concurrent treatment is guided by the occurrence of suitable biomarkers and implemented prior to manifestation of new AD symptoms. Under this strategy, following the initial transient *i*Aβ depletion treatment, no symptomatic AD would recur for the remaining lifetime of the treated individual.

However, in the unlikely case that ACH2.0-based drugs have detrimental long-term administration-associated effects, an alternative strategy can be implemented. In this strategy, the ACH2.0-based drugs are administered transiently but recurrently. Such strategy is illustrated in Figure 18. Figure 18A depicts the outcome of a single administration of an ACH2.0-based *i*Aβ-depleting drug (orange box) in symptomatic unconventional AD with continuously present unconventional stimuli (the worst case scenario). Following the treatment, the levels of *i*Aβ are collapsed, and it can no longer support operation of the AβPP-independent *i*Aβ generation pathway. However, because of the continuous presence of unconventional stimuli (pink box), the AβPP-independent *i*Aβ production pathway remains active. With the withdrawal of the drug, accumulation of *i*Aβ, produced independently of AβPP, resumes, and when it crosses the T1 threshold, the disease recurs. Importantly, even a single transient administration of the drug buys some disease-free time, which is measured probably in years, the time between the withdrawal of the drug and the T1 crossing, when no disease occurs. With the second transient administration of the drug, this time can be doubled, and the treatment can be implemented, recurrently, many times. In this scenario, following the initial transient treatment, the disease would not recur as long as the treatments are repeatedly implemented. This scenario is illustrated in Figure 18B (transient *i*Aβ depletion treatments: orange boxes; continuous presence of unconventional stimuli: pink box). The timing of each following treatment is not significantly restricted by the T1 crossing; the drug can be administered not only before, but also after the crossing (within certain range) with similar effects; ideally, the initial transient treatment should be dispensed either following the asymptomatic biomarkers-based diagnosis of AD or at the earliest symptomatic stage, and each subsequent treatment should be guided by detection of suitable biomarkers and implemented before new or recurrent AD symptoms manifest.

## 16. Targeting the Initial Causes of Unconventional AD

The present section considers the effects of suppression of unconventional (as defined above) stimuli capable of activating and maintaining operation of the AβPP-independent *i*Aβ generation pathway. As reasoned above, for most occurrences and conditions leading to unconventional AD, the stimuli in question apparently include compromised BBB and reduced CBF. The defined nature of the stimuli (other than AβPP-derived *i*Aβ) capable of triggering activation of the eIF2α kinases and eliciting the integrated stress response, and the identity of ISR-unrelated activators of the AβPP-independent *i*Aβ production pathway (provided they occur; discussed in Section 9.4 above) remain to be elucidated. These stimuli, and possibly the ISR-unrelated activators of the AβPP-independent *i*Aβ generation pathway, are referred to here generically as “unconventional stimuli”. Intuitively, it could be assumed that if unconventional stimuli are suppressed or removed prior to commencement of unconventional AD, the disease would not occur. The following two figures illustrate that this is not necessarily the case, but that the long-term suppression of the unconventional stimuli can be very efficient in combination with transient depletion of *i*Aβ by the ACH2.0 drugs. 

### 16.1. Unconventional Stimuli-Suppressing Drugs Can Prevent AD Only in Combination with the Transient iAβ Depletion Treatment

**Figure 19:**
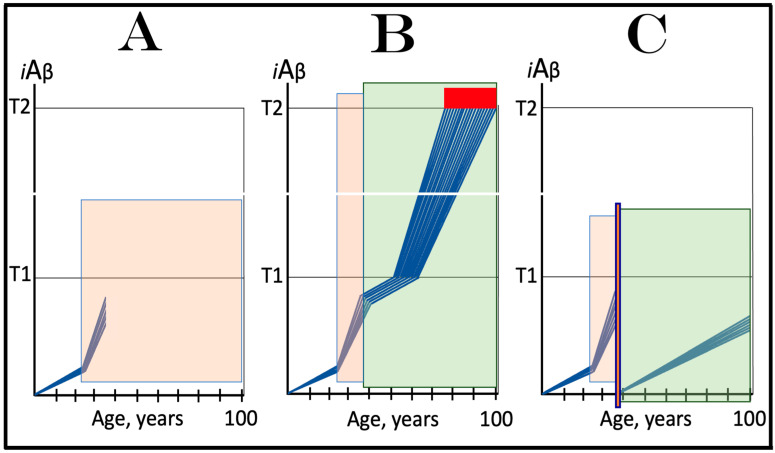
**Unconventional stimuli-suppressing drugs can prevent AD only in combination with the transient *i*Aβ depletion treatment.** *i*Aβ: Intraneuronal Aβ. ***T1*** *threshold*: Cellular concentration of *i*Aβ that triggers, via eIF2α kinases and the integrated stress response, activation of the AβPP-independent *i*Aβ generation pathway. ***T2*** *threshold*: Cellular concentration of *i*Aβ, produced predominantly in the AβPP-independent pathway, which triggers apoptosis or necroptosis of neuronal cells. *Blue lines*: Levels of *i*Aβ in individual neurons. *Red Box*: Apoptotic Zone, the continuum of *i*Aβ levels that lead irreversibly to cell death. *Pink Boxes*: Duration of the presence of unconventional stimulants. *Green Boxes*: Duration of administration of unconventional stimuli-suppressing drug. *Panel* (**A**): The initial state of the levels of *i*Aβ in individual neurons at the commencement of long-term treatment with suppressors of the unconventional stimuli. The AβPP-independent *i*Aβ production pathway has been unconventionally activated by stimuli that would be present for a long-term duration. As the result, *i*Aβ has accumulated substantially, but has not yet reached the T1 threshold. *Panel* (**B**): Evolution of the initial state in the presence of a stimuli-suppressing drug. Since *i*Aβ is below the T1 threshold, it cannot sustain the AβPP-independent *i*Aβ production pathway; with the suppression of the unconventional stimuli, its operation ceases. Accumulation of AβPP-derived *i*Aβ continues from a new high baseline, and when it crosses the T1 threshold, the AβPP-independent *i*Aβ generation pathway is activated and AD commences, unimpeded by the presence of suppressors of the unconventional stimuli. In this scenario, the stimuli-suppressing therapy delays, but does not prevent, the occurrence of the disease. *Panel* (**C**): Evolution of the initial state in the presence of a stimuli-suppressing drug following transient *i*Aβ depletion treatment with an ACH2.0-based drug. The presence of a stimuli-suppressing drug renders the AβPP-independent *i*Aβ production pathway inoperative, whereas transient *i*Aβ depletion treatment with an ACH2.0 drug collapses the levels of *i*Aβ. Following transient depletion of *i*Aβ, its accumulation resumes de novo, supported now only by the AβPP proteolysis. Its levels would not reach the T1 threshold and AD would not occur for the duration of treatment with a stimuli-suppressing drug.

Figure 19 illustrates the effect of unconventional stimuli-suppressing drugs (green boxes) in the prevention of AD. Figure 19A shows the initial state of the levels of *i*Aβ in individual neurons at the commencement of long-term treatment with suppressors of the unconventional stimuli. At this point, due to a certain event or a condition, the AβPP-independent *i*Aβ generation pathway has been unconventionally activated for some time, and would remain operational due to the continuous presence of unconventional stimuli (pink box), and its product (*i*Aβ) has accumulated substantially but is still sub-T1. Figure 19B depicts the evolution of the initial state in the presence of a stimuli-suppressing drug. Since *i*Aβ is below the T1 threshold, it cannot support the operation of the AβPP-independent *i*Aβ production pathway. With the suppression or removal of the unconventional stimuli, operation of the pathway ceases. On the other hand, accumulation of AβPP-derived *i*Aβ continues from a new high baseline. When it reaches and crosses the T1 threshold, the self-sustainable AβPP-independent *i*Aβ generation pathway is activated and AD commences, unimpeded by the presence of suppressors of the unconventional stimuli. In this scenario, the stimuli-suppressing therapy delays, but does not prevent, the occurrence of the disease. Figure 19C illustrates the evolution of the initial state in the presence of a stimuli-suppressing drug following transient *i*Aβ depletion treatment with an ACH2.0-based drug. The presence of a stimuli-suppressing drug renders the AβPP-independent *i*Aβ production pathway inoperative, whereas the transient *i*Aβ depletion treatment with an ACH2.0 drug collapses the levels of *i*Aβ. Following transient depletion of *i*Aβ, its accumulation resumes de novo, supported now only by the AβPP proteolysis. Its levels would not reach the T1 threshold and AD would not occur for the duration of treatment with a stimuli-suppressing drug.

### 16.2. Unconventional Stimuli-Suppressing Drugs Can Be Efficient in Treatment of Symptomatic AD Only If Complemented by the Transient iAβ Depletion Therapy 

**Figure 20:**
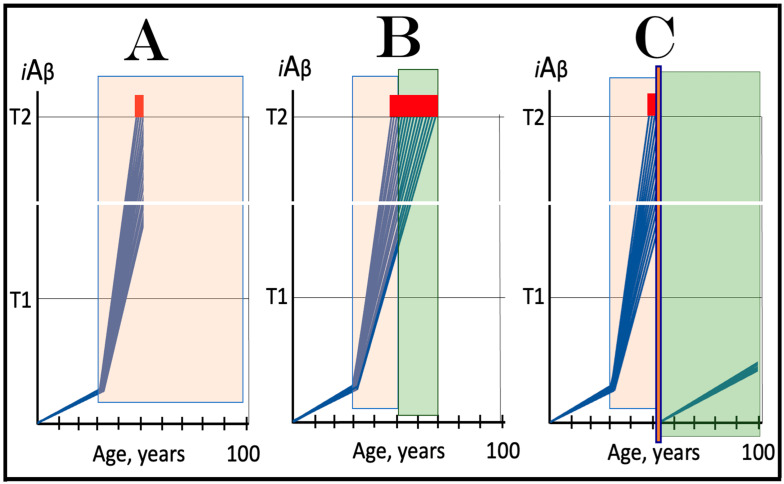
**Unconventional stimuli-suppressing drugs can be effective in unconventional symptomatic AD only in combination with the transient *i*Aβ depletion treatment.** *i*Aβ: Intraneuronal Aβ. ***T1*** *threshold*: Cellular concentration of *i*Aβ that triggers, via eIF2α kinases and the integrated stress response, activation of the AβPP-independent *i*Aβ generation pathway. ***T2*** *threshold*: Cellular concentration of *i*Aβ, produced predominantly in the AβPP-independent pathway, which triggers apoptosis or necroptosis of neuronal cells. *Blue lines*: Levels of *i*Aβ in individual neurons. *Red Box*: Apoptotic Zone, the continuum of *i*Aβ levels that lead irreversibly to cell death. *Pink Boxes*: Duration of the presence of unconventional stimulants. *Green Boxes*: Duration of administration of unconventional stimuli-suppressing drug. *Panel* (**A**): The initial state of the levels of *i*Aβ in individual neurons at the commencement of long-term treatment with suppressors of the unconventional stimuli. The AβPP-independent *i*Aβ production pathway has been unconventionally activated by stimuli that would be present for a long-term duration. *i*Aβ produced independently of AβPP has rapidly accumulated, crossed the T1 threshold, and AD commenced; when *i*Aβ reached the T2 threshold in a fraction of the neurons, AD symptoms manifested. At this point, the AβPP-independent *i*Aβ production pathway is sustained by both *i*Aβ at over-T1 levels and the unconventional stimuli. *Panel* (**B**): Evolution of the initial state in the presence of a stimuli-suppressing drug. The unconventional stimuli are suppressed and can no longer support operation of the AβPP-independent *i*Aβ generation pathway. But the pathway is still sustained by *i*Aβ (since it is over-T1), and remains operational. Therefore, suppression of the unconventional stimuli would have no effect whatsoever on the progression of the disease. *Panel* (**C**): Evolution of the initial state in the presence of a stimuli-suppressing drug following transient *i*Aβ depletion treatment with an ACH2.0-based drug. Suppression of the unconventional stimuli takes away one sustenance source of the AβPP-independent *i*Aβ production pathway whereas transient depletion of *i*Aβ removes another. The AβPP-independent *i*Aβ production is rendered inoperative; *i*Aβ is substantially depleted, and its de novo accumulation, supported only by the AβPP proteolysis, resumes from a low baseline. Its levels would not reach the T1 threshold, and AD would not recur within the remaining lifetime of the individual.

Figure 20 illustrates the effect of the unconventional stimuli-suppressing drugs (green boxes) in the treatment of symptomatic AD. Figure 20A depicts the initial state of the levels of *i*Aβ in individual neurons of a patient at the start of long-term treatment with suppressors of the unconventional stimuli. By this time, the AβPP-independent *i*Aβ generation pathway has been unconventionally activated, with AβPP-derived *i*Aβ levels well below the T1 threshold. *i*Aβ produced independently of AβPP has rapidly (relative to AβPP-derived *i*Aβ) accumulated, crossed the T1 threshold, and AD commenced. *i*Aβ has further accumulated, crossed the T2 threshold in a fraction of the neurons, and AD symptoms manifested. Importantly, at this point the AβPP-independent *i*Aβ production pathway is sustained by both, *i*Aβ (at levels exeeding the T1 threshold) and the unconventional stimuli (pink box). Figure 20B shows evolution of the initial state in the presence of a stimuli-suppressing drug (green box). The unconventional stimuli are suppressed and can no longer support the operation of the AβPP-independent *i*Aβ generation pathway. But the pathway is still sustained by *i*Aβ, since its levels are above the T1 threshold, and therefore it remains operational. Consequently, in this situation, suppression of the unconventional stimuli would have no effect whatsoever on progression of the disease. *i*Aβ, produced independently of AβPP, would continue to accumulate, more neurons would cross the T2 threshold, and the disease would enter its end stage. Figure 20C presents evolution of the initial state in the presence of a stimuli-suppressing drug following the transient *i*Aβ depletion treatment with an ACH2.0-based drug (orange box). Suppression of the unconventional stimuli (green box) takes away one sustenance source of the AβPP-independent *i*Aβ production pathway, whereas transient depletion of *i*Aβ removes another. Without continuous stimulation, operation of the AβPP-independent *i*Aβ production pathway ceases. *i*Aβ is substantially depleted, and its slow de novo accumulation, supported solely by the AβPP proteolysis, resumes from a low baseline. Its levels would not reach the T1 threshold, and AD would not recur (provided the stimuli-suppressing treatment continues) within the remaining lifetime of the individual.

## 17. ISR Inhibitors in Treatment of Conventional and Unconventional AD: Great Potential but Equally Great Possibility of Adverse Effects

With the exception of cases of unconventional AD caused through the activation of the AβPP-independent *i*Aβ generation pathway by ISR-unrelated activators (discussed above), where they would be ineffective, ISR inhibitors hold, apparently, great potential for the treatment of AD. Indeed, in the other two remaining classes of AD, both conventional and unconventional, elicitation of the ISR is the pivotal point in the activation and maintenance of the AβPP-independent *i*Aβ generation pathway. How the ISR is elicited distinguishes between conventional and unconventional AD. In the former, the eIF2α kinases are activated by AβPP-derived *i*Aβ at levels exceeding the T1 threshold, whereas in the latter, stressors distinct from AβPP-derived iAβ activate the eIF2α kinases. In either case, radical re-programming of protein expression under the ISR conditions provides essential components necessary for operation of the AβPP-independent *i*Aβ production pathway and “missing” under regular conditions. Consequently, if the ISR were inhibited and no longer in effect, no required components would be provided, and the AβPP-independent *i*Aβ generation pathway, the Engine that drives the disease, would be rendered inoperative for the duration of the treatment; therefore, to be effective, ISR inhibitors would have to be administered long-term. The effects of such long-term administration of ISR inhibitors in prevention and treatment of both conventional and unconventional AD are considered in the following two figures.

### 17.1. ISR Inhibitors in Prevention and Treatment of Conventional AD 

**Figure 21:**
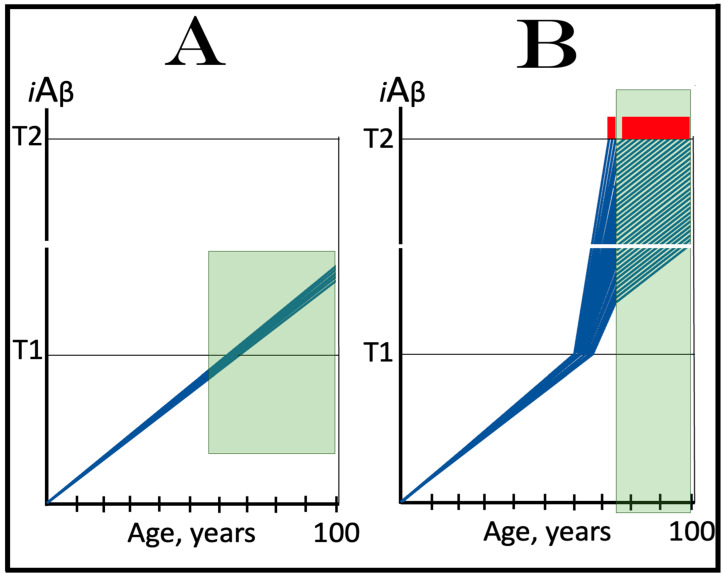
**Inhibitors of the integrated stress response in prevention and treatment of conventional AD.** *i*Aβ: Intraneuronal Aβ. ***T1*** *threshold*: Cellular concentration of *i*Aβ that triggers, via eIF2α kinases and the integrated stress response, activation of the AβPP-independent *i*Aβ generation pathway. ***T2*** *threshold*: Cellular concentration of *i*Aβ, produced predominantly in the AβPP-independent pathway, which triggers apoptosis or necroptosis of neuronal cells. *Blue lines*: Levels of *i*Aβ in individual neurons. *Red Box*: Apoptotic Zone, the continuum of *i*Aβ levels that lead irreversibly to cell death. *Green Boxes*: Duration of administration of the ISR inhibitors. *Panel* (**A**): AβPP-derived *i*Aβ accumulates at such a rate that the crossing of the T1 threshold, activation of the AβPP-independent *i*Aβ production pathway, and commencement of AD are inevitable. Administration of an ISR inhibitor commences when the levels of AβPP-derived *i*Aβ are below the T1 threshold. Inhibition of the ISR does not affect the AβPP proteolytic pathway, and accumulation of AβPP-derived *i*Aβ continues uninterrupted. The effect of the ISR inhibition manifests only with the crossing of the T1 threshold. In the absence of the ISR, the components necessary for operation of the AβPP-independent *i*Aβ generation pathway are not supplied, and the pathway is not activated. Accumulation of *i*Aβ continues at the slow rate, supported only by the AβPP proteolysis. Its levels would not reach the AD pathology-causing range, and AD symptoms would not manifest for the duration of the treatment. *Panel* (**B**): Administration of an ISR inhibitor begins when the AβPP-independent *i*Aβ production pathway has been activated, a fraction of the neurons have crossed the T2 threshold, and AD symptoms manifested. With the ISR suppressed, operation of the AβPP-independent *i*Aβ generation pathway ceases, but the levels of *i*Aβ remain high. Operation of the AβPP proteolytic pathway remains unaffected, and slow accumulation of AβPP-derived *i*Aβ continues. In the presence of ISR inhibitors, *i*Aβ would be crossing the T2 threshold at a much slower rate, but its levels are within the AD pathology-causing range. Consequently, for the duration of the treatment, the disease would be progressing, albeit at much slower rate.

Figure 21 illustrates the effects of ISR inhibitors in the prevention and treatment of conventional AD. In Figure 21A, administration of an ISR inhibitor (green box) commences when the levels of AβPP-derived *i*Aβ are below the T1 threshold. Inhibition of the ISR does not affect the AβPP proteolytic pathway, and accumulation of AβPP-derived *i*Aβ continues uninterrupted. The effect of the ISR inhibition manifests only with the crossing of the T1 threshold. In the absence of the ISR, the components necessary for operation of the AβPP-independent *i*Aβ generation pathway are not supplied, and the pathway is not activated. Accumulation of *i*Aβ continues at the slow rate, supported only by the AβPP proteolysis. Its levels would not reach the AD pathology-causing range, and AD symptoms would not manifest for the duration of the treatment. In Figure 21B, administration of an ISR inhibitor (green box) begins when AβPP-derived *i*Aβ has crossed the T1 threshold, the AβPP-independent *i*Aβ production pathway has been activated, *i*Aβ levels in a fraction of the neurons have crossed the T2 threshold, and AD symptoms have already manifested. With the ISR suppressed, operation of the AβPP-independent *i*Aβ generation pathway ceases, but the levels of *i*Aβ remain high. Operation of the AβPP proteolytic pathway, on the other hand, remains unaffected, and slow accumulation of AβPP-derived *i*Aβ continues. In the presence of ISR inhibitors, *i*Aβ would be crossing the T2 threshold at a much slower rate but its levels are within the AD pathology-causing range. Consequently, for the duration of the treatment, the disease would be progressing, albeit at much slower rate. It should be commented that, due to the absence of unconventional stimuli, concurrent utilization of the transient *i*Aβ depletion therapy and ISR suppressors is inapplicable in conventional AD. This is because the former, i.e., transient iAβ depletion treatment alone, would be fully sufficient in this case (as depicted in Figure 13 above) and the implementation of the latter would be unnecessary. However, as described in Section 17.2 below, such combined therapy could be fruitful in treatment of unconventional AD. 

### 17.2. Effect of ISR Inhibitors in Prevention and Treatment of Unconventional AD

**Figure 22:**
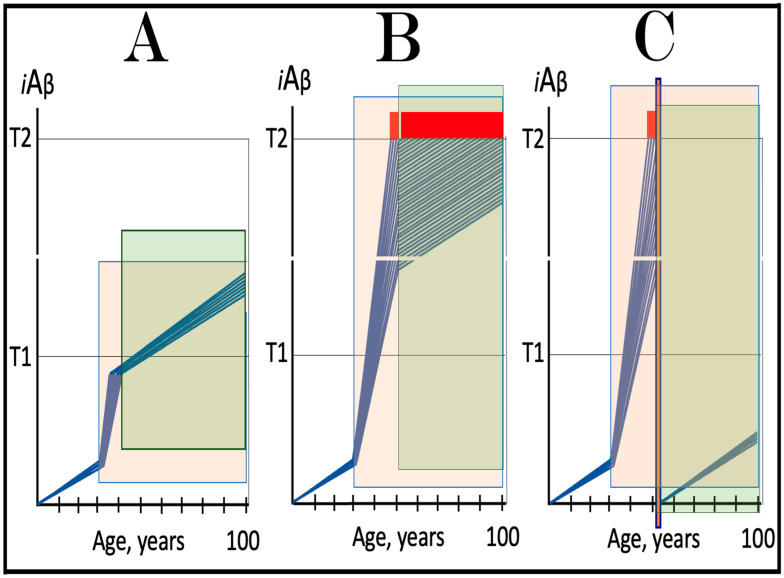
**The ISR inhibitors in prevention and treatment of unconventional AD.** *i*Aβ: Intraneuronal Aβ. ***T1*** *threshold*: Cellular concentration of *i*Aβ that triggers, via eIF2α kinases and the integrated stress response, activation of the AβPP-independent *i*Aβ generation pathway. ***T2*** *threshold*: Cellular concentration of *i*Aβ, produced predominantly in the AβPP-independent pathway, which triggers apoptosis or necroptosis of neuronal cells. *Blue lines*: Levels of *i*Aβ in individual neurons. *Red Box*: Apoptotic Zone, the continuum of *i*Aβ levels that lead irreversibly to cell death. *Pink Boxes*: Duration of the presence of unconventional stimulants. *Green Boxes*: Duration of administration of the ISR inhibitors. *Panel* (**A**): The ISR suppression treatment commences after the AβPP-independent *i*Aβ generation pathway has been unconventionally activated, but before the T1 crossing. With the ISR suppressed, operation of the AβPP-independent *i*Aβ production pathway ceases. The treatment does not affect the AβPP proteolysis. AβPP-derived *i*Aβ continues to accumulate from a high baseline and its levels cross the T1 threshold. In the absence of the ISR, with the necessary components unavailable, the AβPP-independent *i*Aβ production pathway remains inoperative despite the over-T1 levels of AβPP-derived *i*Aβ. Continuous accumulation of *i*Aβ is sustained solely by the AβPP proteolysis and proceeds at slow rate; its levels would not reach the AD pathology-causing range, and AD symptoms would not occur for the duration of the treatment. *Panel* (**B**): When the ISR suppression treatment starts, *i*Aβ levels in a fraction of the neurons have already crossed the T2 threshold, and AD symptoms have manifested. Suppression of the ISR renders the AβPP-independent *i*Aβ generation pathway inoperative, but the levels of *i*Aβ remain high, and its accumulation, sustained by the AβPP proteolysis, continues. The T2 crossing would occur at a much slower rate, but the levels of *i*Aβ would remain within the AD pathology-causing range and the disease would progress, although slower than in the absence of the drug. *Panel* (**C**): The same situation as in panel (**B**), but concurrently with commencement of the ISR suppression, the transient *i*Aβ depletion treatment is implemented. With *i*Aβ depleted and with unconventional stimuli ineffective due to the ISR inhibition, operation of the AβPP-independent *i*Aβ production pathway ceases. Accumulation of AβPP-derived *i*Aβ resumes from a low baseline; its levels would not reach the T1 threshold, and AD would not recur for the duration of the ISR suppression treatment.

The effects of ISR inhibitors in the prevention and treatment of unconventional AD are illustrated in Figure 22. In panel A, the treatment (green box) commences after the AβPP-independent *i*Aβ generation pathway has been unconventionally activated (the duration of the presence of unconventional stimuli is denoted by the pink box), but before the T1 crossing. With the ISR suppressed, the operation of the AβPP-independent *i*Aβ production pathway ceases. The treatment does not affect the AβPP proteolysis. AβPP-derived *i*Aβ continues to accumulate from a high baseline, and its levels cross the T1 threshold. In the absence of the ISR, with its necessary components unavailable, the AβPP-independent *i*Aβ production pathway remains inoperative despite the over-T1 levels of AβPP-derived *i*Aβ. Continuous accumulation of *i*Aβ is sustained solely by the AβPP proteolysis and proceeds at slow rate; its levels would not reach the AD pathology-causing range, and AD symptoms would not occur for the duration of the treatment. In Figure 22B, *i*Aβ produced in the unconventionally activated AβPP-independent pathway crosses the T1 threshold (pink box). When the ISR suppression treatment (green box) starts, *i*Aβ levels in a fraction of the neurons have already crossed the T2 threshold, and AD symptoms have manifested. Suppression of the ISR renders the AβPP-independent *i*Aβ generation pathway inoperative, but the levels of *i*Aβ remain high, and the continuous slow accumulation of *i*Aβ is sustained by the AβPP proteolysis. The T2 crossing would occur at a much slower rate, but the levels of *i*Aβ would remain within the AD pathology-causing range, and the disease would progress, although slower than in the absence of the drug. Figure 22C considers the same situation as panel B, but with a single change: concurrently with commencement of the ISR suppression, the transient *i*Aβ depletion treatment is implemented (orange box). With *i*Aβ depleted and with unconventional stimuli ineffective due to ISR inhibition, operation of the AβPP-independent *i*Aβ production pathway ceases. Accumulation of AβPP-derived *i*Aβ resumes from a low baseline; its levels would not reach the T1 threshold and AD would not recur for the duration of the ISR suppression treatment.

### 17.3. The Possibility of Adverse Effects Associated with the Long-Term Administration of ISR Inhibitors Is Substantial

It should be emphasized, however, that the possibility of adverse effects associated with the long-term administration of ISR inhibitors is no less great than their therapeutic potential. Indeed, the ISR is a basic cellular survival tool, and it is hard to imagine that its systemic elimination or weakening would not adversely affect the organism. That this is indeed the case is strongly indicated by observations with transgenic eIF2α Ser51Ala KI mice. In elicitation of the ISR, the point of “integration”, i.e., of the convergence of various stressors, is the phosphorylation of eIF2α at its serine residue 51. This causes cellular transcriptional and translational re-programming and ushers in the ISR. It follows that if this phosphorylation event were suppressed, no ISR would be elicited. This is exactly what is accomplished by the serine 51 to alanine substitution in eIF2α. Ala51 cannot be phosphorylated by the eIF2α kinases and, consequently, the IRS cannot be elicited in the carriers of this substitution. Thus, the effect of the prevention of elicitation of the ISR in transgenic mice forecasts what can be expected in human cases where the ISR is systemically inhibited. This forecast is not promising: mice carrying a homozygous Ser51Ala mutation did not survive past 18 h after their birth [230]. It can be argued that partial long-term inhibition of the ISR could potentially be a solution. When an adequate transgenic AD model is available (reviewed in [7]), this approach can and should be tested; it would not be surprising, however, if it turns out to be unproductive.

## 18. Conclusions: The ACH2.0/E Is a Unified Alzheimer’s Field Theory

### 18.1. Concept of “Unconventional” Alzheimer’s Disease Is the Inevitable Expansion of Logic of the ACH2.0 Theory of Conventional AD

#### 18.1.1. Conventional Alzheimer’s Disease

Formulation of the ACH2.0 was necessitated by two principal factors. One was the accumulated empirical data that made it clear that extracellular Aβ neither causes nor drives AD. Another principal factor was the equally persuasive data, including the observations that, without a single exception, all known numerous mutations associated with the disease affect either the production and intraneuronal degradation or structure of Aβ, and thus indicate the unquestionable centrality of Aβ in AD. In the ACH2.0 the roles of the causative agent and the driver of the disease are assumed by differentially derived intraneuronal Aβ, *i*Aβ. The ACH2.0 posits that when AβPP-derived *i*Aβ accumulates over the critical threshold (the T1 threshold), it triggers AD, a notion supported by the observation that all factors predisposing to or causing the disease increase the rate of AβPP-derived *i*Aβ accumulation, whereas factors protecting against the occurrence of AD reduce it. Triggering AD entails activating the AβPP-independent *i*Aβ generation pathway. The bridge between AβPP-derived *i*Aβ and the AβPP-independent production of *i*Aβ is the neuronal integrated stress response, ISR. AβPP-derived *i*Aβ, at the over-T1 levels, triggers activation of PKR and/or HRI, eIF2α gets phosphorylated at serine residue 51, and the neuronal ISR is elicited. The ensuing radical transcriptional and translational re-programming provides the essential components, which are necessary for the operation of the AβPP-independent *i*Aβ generation pathway but are “missing” under the regular conditions, and the pathway is activated. Its entire *i*Aβ output is retained intraneuronally. *i*Aβ rapidly accumulates and fulfills two key functions: it drives the AD pathology and propagates the neuronal ISR, thus perpetuating its own production in the AβPP-independent manner. The AβPP-independent *i*Aβ production pathway constitutes the active core of AD, the AD “Engine”, and the described sequence of events is defined as “conventional” AD [1,2,3,4,5,6,7].

#### 18.1.2. Unconventional Alzheimer’s Disease

The above reasoning makes it inevitable that another class of the disease, “unconventional” AD, has to occur. This is because the ISR, this pivotal bridge to the active core of AD, can be elicited in neuronal cells by a multitude of stressors distinct from AβPP-derived *i*Aβ. As described above, these stressors can be provided by multiple occurrences and conditions long associated with AD, such as traumatic brain injury, chronic traumatic encephalopathy, viral and bacterial infections, and numerous inflammatory conditions. Most, if not all, of these have two common features: they compromise the blood–brain barrier and reduce cerebral blood flow. The former provides plentiful opportunities for stressors capable of activating the eIF2α kinases to penetrate the brain and, subsequently, the neurons. The latter is capable of triggering mitochondrial distress in neuronal cells. This leads, via the OMA1 to the DELE1 signaling pathway, to activation of HRI. In either case, eIF2α is phosphorylated, the neuronal ISR is elicited, the AβPP-independent *i*Aβ generation pathway is activated, and unconventional AD eventually ensues. Importantly, both forms of AD, conventional and unconventional, are identical in their “active core”, i.e., the AβPP-independent *i*Aβ production pathway, and differ only in the manner of their causation. Conventional AD is caused by AβPP-derived *i*Aβ accumulated over the T1 threshold. Unconventional AD is caused by “unconventional” stimuli distinct from AβPP-derived *i*Aβ. Importantly, in its both conventional and unconventional forms, the disease is driven, from the instance of its commencement, by *i*Aβ generated independently of AβPP. The combined notions of conventional and unconventional AD constitute the expanded ACH2.0, the ACH2.0/E theory of AD. To summarize, the ACH2.0/E defines the operational, self-sustainable, disease-driving core, namely, the AβPP-independent *i*Aβ generation pathway common to both forms of AD, and identifies the causative pathways, which activate this core in a disease form-specific manner. By virtue of consolidating the intertwined aspects into a single mechanistic entity, the ACH2.0/E constitutes a unified Alzheimer’s field theory. 

#### 18.1.3. Two Principal Distinctions between Conventional and Unconventional Forms of Alzheimer’s Disease

As discussed above, one principal distinction between conventional and unconventional forms of AD is the manner of causation of the disease. In the former, the causative agent is AβPP-derived *i*Aβ accumulated physiologically over the T1 threshold whereas in the latter it is a stressor distinct from *i*Aβ and capable of eliciting the neuronal ISR. Another principal distinction between the two forms of AD is the relationship between activity of the AβPP-independent *i*Aβ generation pathway and the disease. In conventional AD, activation of the AβPP-independent *i*Aβ production pathway is synonymous with commencement of the disease. This is because, at this stage, levels of *i*Aβ exceed the T1 threshold and the pathway is self-sustainable from the moment of its inception; cessation of the influx of the initial ISR-eliciting stimulus, i.e., AβPP-derived *i*Aβ, would be completely inconsequential at this point.

In sharp contrast, in unconventional AD, elicitation of the neuronal ISR and subsequent activation of the AβPP-independent *i*Aβ generation pathway always occurs at the levels of *i*Aβ below the T1 threshold (otherwise it would be a typical case of conventional AD). Consequently, until and unless the T1 threshold is crossed, the AβPP-independent *i*Aβ production pathway is not self-sustainable; if the presence of an unconventional ISR-eliciting stimulus were to cease, so would the operation of the pathway. The self-sustainability is attained with the T1 crossing, when the levels of *i*Aβ become sufficient to propagate the ISR, and thus to perpetuate operation of the AβPP-independent pathway of its own production; only at this point does the disease commence. Transient operation of the unconventionally activated AβPP-independent *i*Aβ generation pathway, terminating prior to the T1 crossing, is, however, not benign. It elevates the *i*Aβ baseline, and thus accelerates the T1 crossing and hastens the occurrence of AD. This is apparently how TBI suffered by an athlete in his 20s results in AD in his 40s, and how stepwise elevation of *i*Aβ levels, due to recurrent transient operation (“pulses of activity”) of the unconventionally activated AβPP-independent *i*Aβ production pathway, leads to AD in CTE cases (both scenarios are depicted diagrammatically in Figure 8 above).

#### 18.1.4. Conventional Alzheimer’s Disease Can Morph into the Unconventional Form

Conceivably, possibly even commonly, following the conventional commencement of Alzheimer’s disease, an event can occur or condition can develop (i.e., TBI, CTE, viral or bacterial infection, neuroinflammation, or systemic inflammation) that results in the occurrence in neuronal cells of unconventional (i.e., distinct from AβPP-derived *i*Aβ) stressors capable of eliciting the ISR. Consequently, due to the presence of unconventional stimulants, the disease would convert from conventional to unconventional. Neither its nature nor its progression would be affected by this morphing; operationally, the disease would continue to be driven by *i*Aβ produced independently of AβPP. The main consequence of such conversion would be practical, namely, the design of the therapeutic strategy. In conventional AD, a single implementation of the transient *i*Aβ depletion therapy could be sufficient for treatment and cure of the disease because the AβPP-independent *i*Aβ generation pathway would be disabled for the remaining lifetime of a patient. In unconventional AD, on the other hand, following depletion of *i*Aβ, the activity of AβPP-independent *i*Aβ production pathway would be sustained via maintenance of the neuronal ISR by unconventional stimulants. In such a case, to prevent restoration of *i*Aβ to AD pathology-driving levels, the *i*Aβ depletion therapy should be implemented either long-term or transiently but recurrently, guided by the occurrence of the relevant biomarkers. The bottom line is that unless the type, i.e., conventional versus unconventional, of the disease were determined, it should be treated as the latter.

### 18.2. iAβ-Depleting Drugs, Such as BACE1 and BACE2 Activators, Are Apparently the Most Versatile Agents for Prevention and Treatment of Both Conventional and Unconventional Forms of Alzheimer’s Disease

The present study discusses various therapeutic strategies and different classes of drugs for both conventional and unconventional AD. One such class consists of ACH-based drugs. These are drugs that either reduce the levels of extracellular Aβ or suppress Aβ production in the AβPP proteolytic pathway. By doing this, they can reduce and possibly even reverse the rate of accumulation of AβPP-derived *i*Aβ; this would delay or prevent both the T1 crossing and the occurrence of AD. However, the ACH-based drugs can have no effect whatsoever in symptomatic AD because the AβPP-independent *i*Aβ generation pathway is completely unresponsive to them. Moreover, these drugs are inapplicable in unconventional AD, where stimuli other than AβPP-derived *i*Aβ elicit the ISR and trigger activation of the AβPP-independent *i*Aβ production pathway. On the other hand, the ACH2.0-based *i*Aβ-depleting drugs, administered only transiently, are capable of preventing conventional AD and effectively treating it at its symptomatic stages. Moreover, the ACH2.0-based drugs are fully competent at preventing unconventional AD and treating it at the symptomatic stages if administered long-term (as shown in Figure 17) or transiently and recurrently (as depicted is Figure 18 above). In contrast, suppressors of the unconventional stimuli (i.e., stimuli capable of eliciting the ISR and thus activating and maintaining the AβPP-independent *i*Aβ generation pathway) could be effective in preventing and treating unconventional AD only if implemented in conjunction with the transient *i*Aβ depletion treatment with ACH2.0-based drugs. As for the ISR inhibitors, they are, apparently, capable of preventing both conventional and unconventional AD, but have limited efficiency in conventional symptomatic AD and require concurrent transient administration of an ACH2.0-based drug for treatment of the disease at the symptomatic stages. It appears, therefore, that the ACH2.0-based *i*Aβ-depleting drugs are by far the most versatile agents for the prevention and treatment of both conventional and unconventional forms of AD and the only type of drugs capable of doing this on its own. It follows that the main therapeutic thrust in Alzheimer’s disease should be directed towards development of the ACH2.0-based drugs, i.e., the agents, such as the activators of the intra-*i*Aβ-cleaving abilities of BACE1 and BACE2 (discussed in Section 13 above), capable of *i*Aβ depletion via its targeted degradation.

### 18.3. Unconventional Activation of the AβPP-Independent iAβ Production Pathway Can Cause Aging-Associated Cognitive Decline (AACD)

In previous studies [4,6] we posited that AACD occurs in individuals with relatively high extends of T1 threshold (apparently the majority of the population) and is caused by AβPP-derived *i*Aβ at the levels below the T1 but over a certain threshold designated T^0^ [4,6]. This description applies in situations where the AβPP-independent *i*Aβ generation pathway is activated conventionally, i.e., by AβPP-derived *i*Aβ accumulated over the T1 threshold. In terms of the present study the picture is more complex because AACD can be triggered also by *i*Aβ produced in the unconventionally activated AβPP-independent *i*Aβ production pathway initiated below the T1 threshold. AACD has been defined operationally as neurodegeneration caused by *i*Aβ at concentrations between the T^0^ and T1 thresholds [4,6]. Accordingly, as soon as the T^0^ threshold is crossed (either by AβPP-derived *i*Aβ or by *i*Aβ produced in the AβPP-independent pathway initiated unconventionally below the T1 threshold), AACD would commence and continue either for the remaining lifespan, if the T1 threshold is not reached, or until the T1 ctrossing; with the crossing of the T1 threshold it would morph into AD. The nature of AACD triggered by *i*Aβ produced in the unconventionally activated AβPP-independent pathway would be identical to that of AACD activated by AβPP-derived *i*Aβ, but applicable therapeutic options would differ significantly. In AACD initiated by AβPP-derived *i*Aβ, ACH-based drugs would be capable of protection from or treating the condition by lowering or reversing the rate of accumulation of AβPP-derived *i*Aβ [4,6]. On the other hand, in AACD triggered by *i*Aβ produced in the unconventionally activated AβPP-independent pathway, ACH-based drugs would be completely ineffective for the reasons discussed above. Nevertheless, regardless of the origins of AACD, ACH2.0-based *i*Aβ depleting drugs would be effective both preventively and curatively. In conventionally initiated AACD, a single transient *i*Aβ depletion treatment could suffice [4,6] whereas in unconventionally triggered AACD, a long-term *i*Aβ depletion therapy or its transient but recurrent applications are expected to be highly efficient.

### 18.4. Conventional AD Apparently Contains, Intrinsically, the Seeds of Unconventional Alzheimer’s Disease

The analysis imparted in the present study opens up a tantalizing possibility, namely, that conventional AD may contain, intrinsically, the seeds of unconventional Alzheimer’s disease. The rationale for this notion is as follows. Above, we reasoned that certain occurrences (e.g., TBI) or conditions (e.g., infection, inflammation) capable of triggering unconventional activation of the AβPP-independent *i*Aβ generation pathway all have two common features: compromised BBB, and reduced CBF. The former provides plentiful opportunities for unconventional stimuli, which are capable of triggering activation of AβPP-independent *i*Aβ generation pathway, to penetrate the brain and, subsequently, the neurons, whereas the latter causes mitochondrial dysfunction in the neurons and triggers the activation of HRI and, possibly, of other eIF2α kinases by “unconventional” means distinct from AβPP-derived *i*Aβ (the only “conventional” trigger of the activation of eIF2α kinases in conventional AD). In either case, the AβPP-independent *i*Aβ production pathway is unconventionally activated, and, consequently, unconventional AD ensues. 

But now consider conventional AD. It is caused by and commences at the levels of AβPP-derived *i*Aβ above the T1 threshold, in the absence of other, unconventional, stimuli. Yet, as the disease progresses, it apparently triggers the very same features mentioned above, namely, compromised BBB and reduced CBF, both presumably associated with, among other factors, the deposition of extracellular Aβ around blood vessels, and both constituting “unconventional stimuli” potentially facilitating unconventional activation of the AβPP-independent *i*Aβ generation pathway. In other words, at this point, the AβPP-independent *i*Aβ production pathway (and the disease) has two sources of self-propagation, namely, *i*Aβ at the over-T1 levels and the unconventional stimuli. It follows that if and when *i*Aβ is depleted by transient administration of an ACH2.0-based drug and can no longer support and propagate the operation of the AβPP-independent *i*Aβ production pathway, the pathway remains, nevertheless, active due to the presence of the unconventional stimuli (generated by and in the course of conventional AD), which take over the pathway’s propagation and maintenance and thus render the disease “unconventional”. It further follows that a single transient *i*Aβ depletion therapy may be insufficient on its own for treatment of conventional AD (it would provide only a temporary relief until *i*Aβ, produced in the now unconventionally sustained AβPP-independent pathway, reaches the T1 threshold and the disease recurs (as shown in Figure 18A), and that ether it should be implemented recurrently (as shown in Figure 18B), or the long-term *i*Aβ depletion treatment (as depicted in Figure 17) should be employed for this purpose. Alternatively, as discussed above, a single transient administration of the *i*Aβ depletion treatment could be combined with the long-term suppression of unconventional stimuli or with the inhibition of the ISR. It follows, in other words, that at its symptomatic stages, conventional Alzheimer’s disease may have to be treated as the unconventional one. 

Moreover, it could be argued that it is not conventional AD that causes the reduction in CBF and compromises BBB but, reversely, that the deposition of extracellular Aβ around the blood vessels, in conjunction with other factors, compromises BBB and reduces CBF, which, in turn, causes (unconventionally) the majority of AD cases by either allowing for the penetration of unconventional stressors or activating, via mitochondrial dysfunction, the HRI kinase and, consequently, phosphorylating eIF2α, eliciting the neuronal ISR, initiating the AβPP-independent *i*Aβ production pathway, and thus commencing the disease. If this were the case, “conventional” AD, as defined above, may be only a minority of cases, and the main category of Alzheimer’s disease could be “unconventional”. Such an interpretation of the etiology of AD currently appears unlikely, but even if it were correct, the therapeutic strategies outlined above, such as the long-term administration of the *i*Aβ-depleting AD drugs or their recurrent transient implementation would remain fully effective both preventively and curatively. 

### 18.5. Validation of the Concept and Evaluation of Potential AD Drugs

The above notions can and must be tested in an adequate animal model of AD, which is currently unavailable (reviewed in [7]). Design and construction of such a model is described in detail in [7]. In the meantime, the concepts articulated above can be tested in a human neuronal cells-based model of AD [231]. This model was constructed with the aim to maximize deposition of extracellular Aβ. Although not intended by the authors of [231], the model’s design facilitated intraneuronal accumulation of exogenously produced AβPP-derived *i*Aβ. Indeed, this model overexpresses exogenously human AβPP carrying the London (V717I) and Swedish (K670N/M671L) mutations; in addition, it expresses exogenously PSEN1 carrying the ΔE9 FAD mutation. As discussed above and elsewhere [7], the London and PSEN1 ΔE9 mutations facilitate production and secretion of the Aβ42 isoform, which is taken up (secreted Aβ does not diffuse in the semi-solid matrigel medium) by the cells at the rate twice that of other Aβ isoforms, whereas the Swedish mutation expedites gamma-cleavage of C99 on intracellular membranes and thus promotes retention of the resulting *i*Aβ. As the result, in the ACH2.0 perspective, AβPP-derived *i*Aβ rapidly accumulates, crosses the T1 threshold, elicits the ISR, and activates the endogenous AβPP-independent *i*Aβ generation pathway; this drives the cellular AD pathology, and its principal hallmark, NFT, is formed (reviewed and interpreted in [7]). The utility of this model in validation of the ACH2.0, principally via the detection of C100 (N-terminal Met-C99) and/or of N-terminal Met-Aβ, has been described elsewhere [4,7]. In terms of the ACH2.0/E, cellular AD pathology can be induced, and the formation of NFTs can be triggered in these cells either through exogenous expression of retainable *i*Aβ [7] or via sustainable elicitation of the ISR by stressors distinct from *i*Aβ [7], and both (cellular AD pathology and NFT formation) can be prevented in the presence of the ISR inhibitor ISRIB. They can also be prevented by exogenous overexpression of BACE1 and/or BACE2. Such results, if obtained, would validate the principal tenets of the ACH2.0/E as well as the proposed therapeutic strategy for Alzheimer’s disease. Moreover, this experimental system could serve as a platform for the development and initial evaluation of potential AD drugs.

## Data Availability

The original contributions presented in the study are included in the article, further inquiries can be directed to the corresponding author/s.
